# Correction: A kinase-dependent checkpoint prevents escape of immature ribosomes into the translating pool

**DOI:** 10.1371/journal.pbio.3000960

**Published:** 2020-10-13

**Authors:** Melissa D. Parker, Jason C. Collins, Boguslawa Korona, Homa Ghalei, Katrin Karbstein

## Notice of Republication

This article was republished on October 12, 2020, to correct an error in Fig 4D, in which the labels “+e.v.” and “+ Rio1” had been switched. Please download this article again to view the correct version. The originally published, uncorrected article and the republished, corrected articles are provided here for reference.

## Supporting information

S1 File Originally published, uncorrected article. (PDF)

S2 File Republished, corrected article. (PDF)
