# Supplementary figures and images for: A kinase-dependent checkpoint prevents escape of immature ribosomes into the translating pool
Source: PLoS Biol. 2019 Dec 13;17(12):e3000329. doi: 10.1371/journal.pbio.3000329 (PMC6934326; doi:10.1371/journal.pbio.3000329)

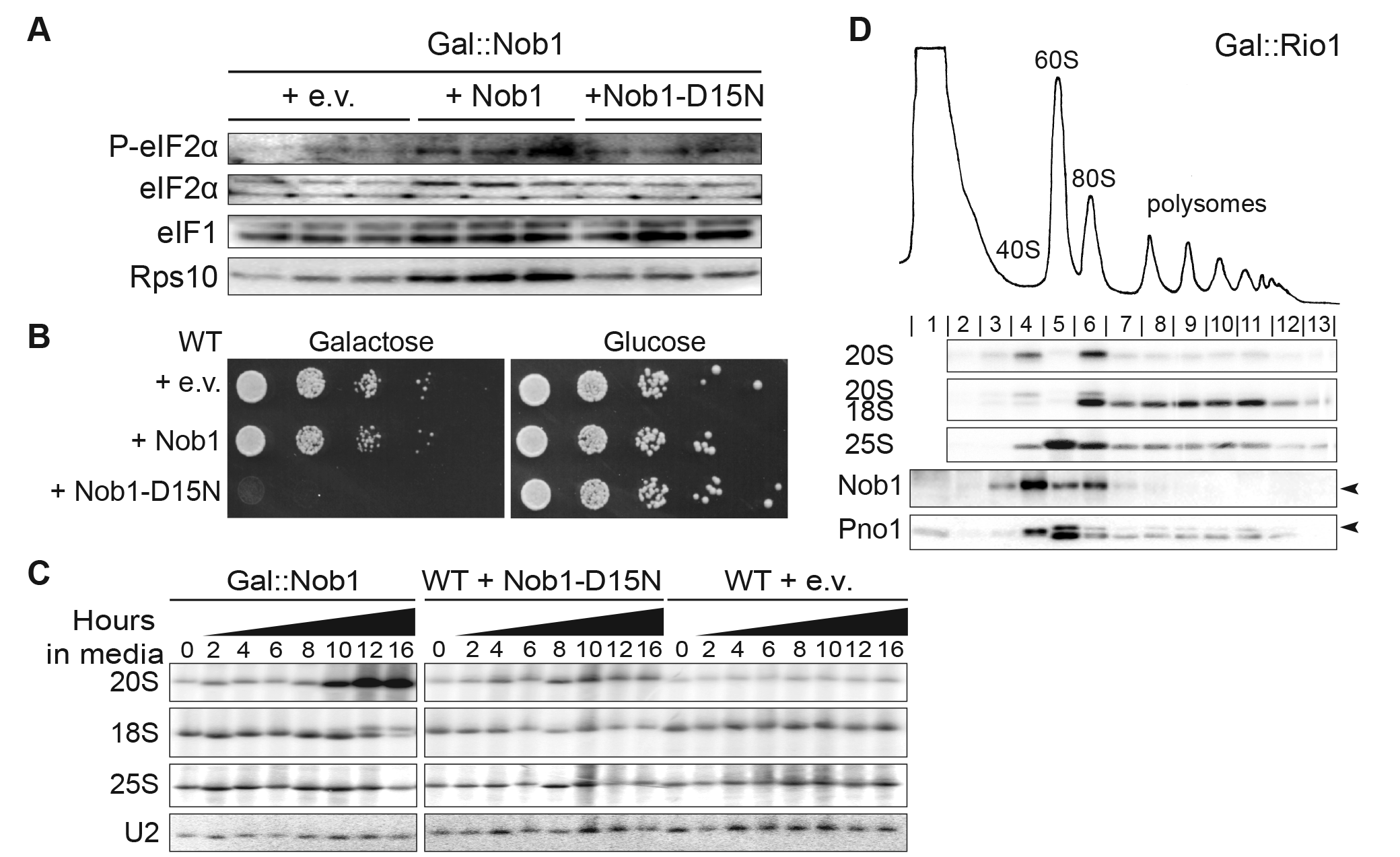

Supplement: S1 Fig — (A) Depletion of Nob1 or expression of Nob1-D15N does not induce the cellular stress response. Western blots from total yeast cell lysates, probed for the indicated proteins. (B) Growth of WT yeast cells transformed with an e.v. or Nob1 or Nob1-D15N under the galactose-inducible, glucose-repressible Gal promoter were compared by 10-fold serial dilutions on glucose or galactose dropout plates. (C) Northern blot analyses of total cellular RNA from cells depleted of Nob1 grown in glucose for the indicated times and total cellular RNA from WT BY4741 cells overexpressing Nob1-D15N or transformed with an e.v. grown in galactose for the indicated times. (D) Shown are 10%–50% sucrose gradient from cell lysate of Tsr1-TAP; Gal::Rio1 cells depleted of Rio1 by growth in YPD for 16 h. Northern blots of 20S, 18S, and 25S rRNA and western blots probing for Nob1 and Pno1 are shown below the absorbance profile at 254 nm. Arrowheads note the bands corresponding to Nob1 and Pno1. Most 20S rRNA accumulated in 80S-like ribosomes (fraction 6). e.v., empty vector; WT, wild-type. (TIF) [file pbio.3000329.s001.tif]

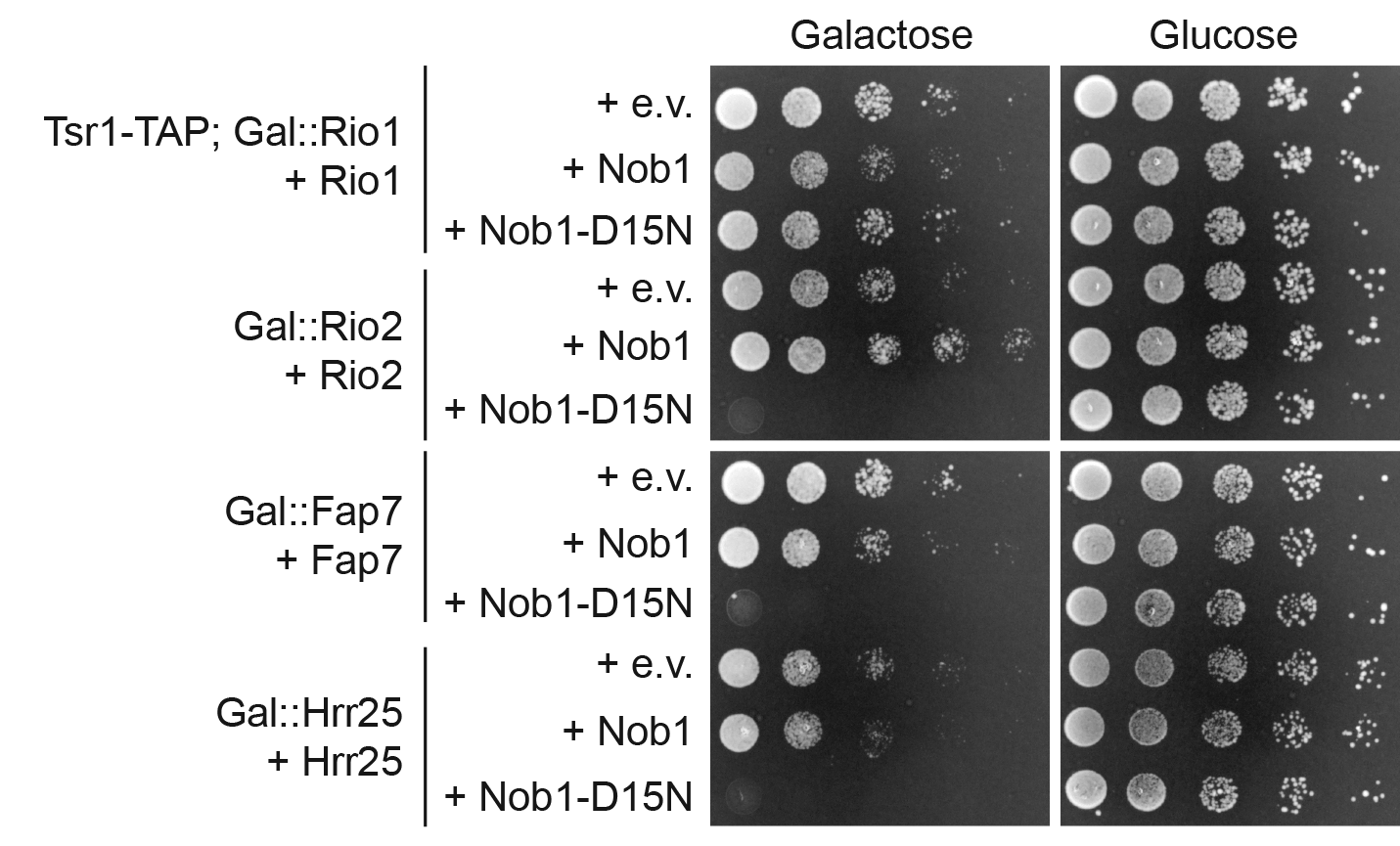

Supplement: S2 Fig — Growth of the indicated cells containing an empty vector or Nob1 or Nob1-D15N under the Gal promoter were compared by 10-fold serial dilutions on galactose or glucose dropout plates. Gal, galactose. (TIF) [file pbio.3000329.s002.tif]

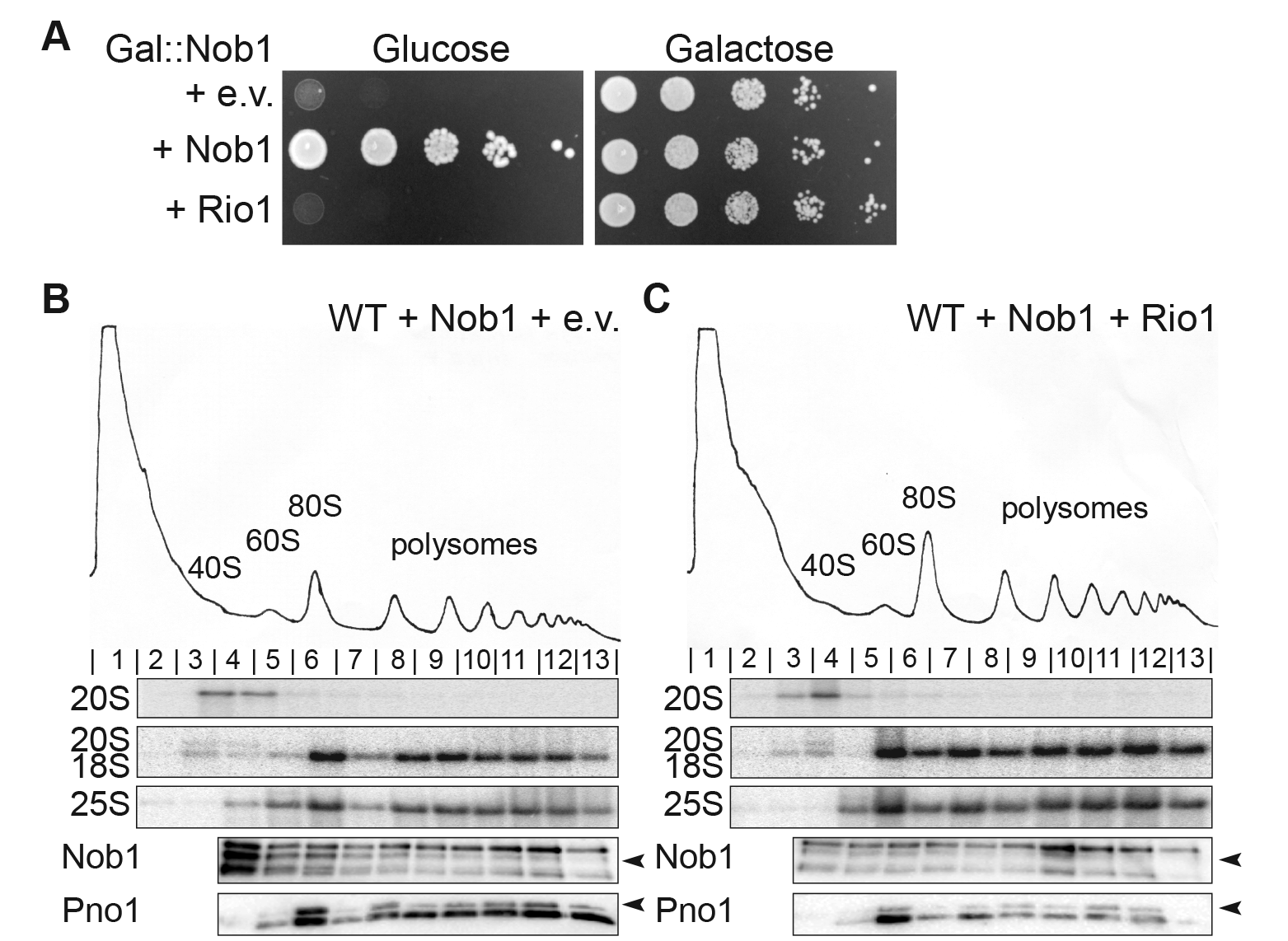

Supplement: S3 Fig — (A) Overexpression of Rio1 does not rescue Nob1 depletion. Growth of cells containing Nob1 under a Gal promoter and expressing either Nob1 or Rio1 from a plasmid under a copper-inducible (Cup1) promoter or an empty vector were compared by 10-fold serial dilutions on glucose or galactose dropout plates with 100 μM CuSO4. (B, C) Sucrose gradient from wild-type cells transformed with an empty vector and overexpressing wild-type Nob1 under a Gal promoter grown in galactose with 100 μM CuSO4 for 16 h. Shown below the absorbance profile at 254 nm are northern blots of 20S, 18S, and 25S rRNAs and western blots probing for Nob1 and Pno1. Arrowheads note the bands corresponding to Nob1 and Pno1. Gal, galactose. (TIF) [file pbio.3000329.s003.tif]

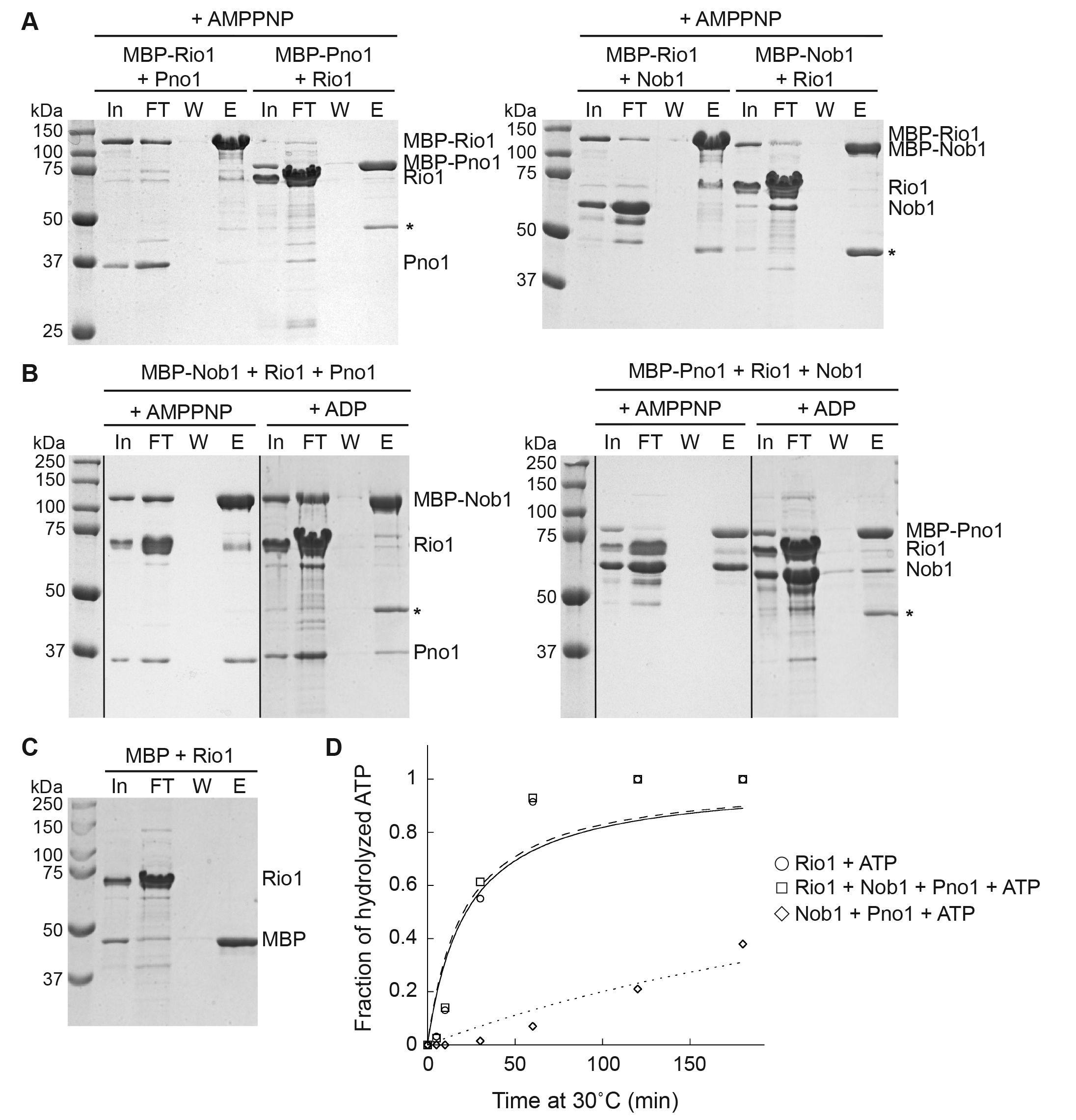

Supplement: S4 Fig — (A) Rio1 does not bind Nob1 or Pno1 individually. Shown are Coomassie-stained SDS-PAGE gels of protein binding assays of purified, recombinant MBP-Rio1, Rio1, MBP-Nob1, Nob1, MBP-Pno1, and Pno1 in the presence of AMPPNP. (B) Coomassie-stained SDS-PAGE gels of protein binding assays on amylose beads of purified, recombinant MBP-Nob1, Nob1, MBP-Pno1, Pno1, and Rio1 in the presence of AMPPNP or ADP. The order of the samples was edited for clarity. (C) Rio1 does not bind MBP. Shown is a Coomassie-stained SDS-PAGE gel of a protein binding assay of purified, recombinant MBP and Rio1. Nob1 and Pno1 also do not bind MBP alone [18]. *MBP. (D) Addition of Nob1 and Pno1 (squares) does not increase the rate of ATP hydrolysis by Rio1 (circles). Numerical data are listed in S1 Data. AMPPNP, adenylyl-imidodiphosphate; E, elution; FT, flow through; In, input; MBP, maltose-binding protein; W, final wash. (TIF) [file pbio.3000329.s004.tif]

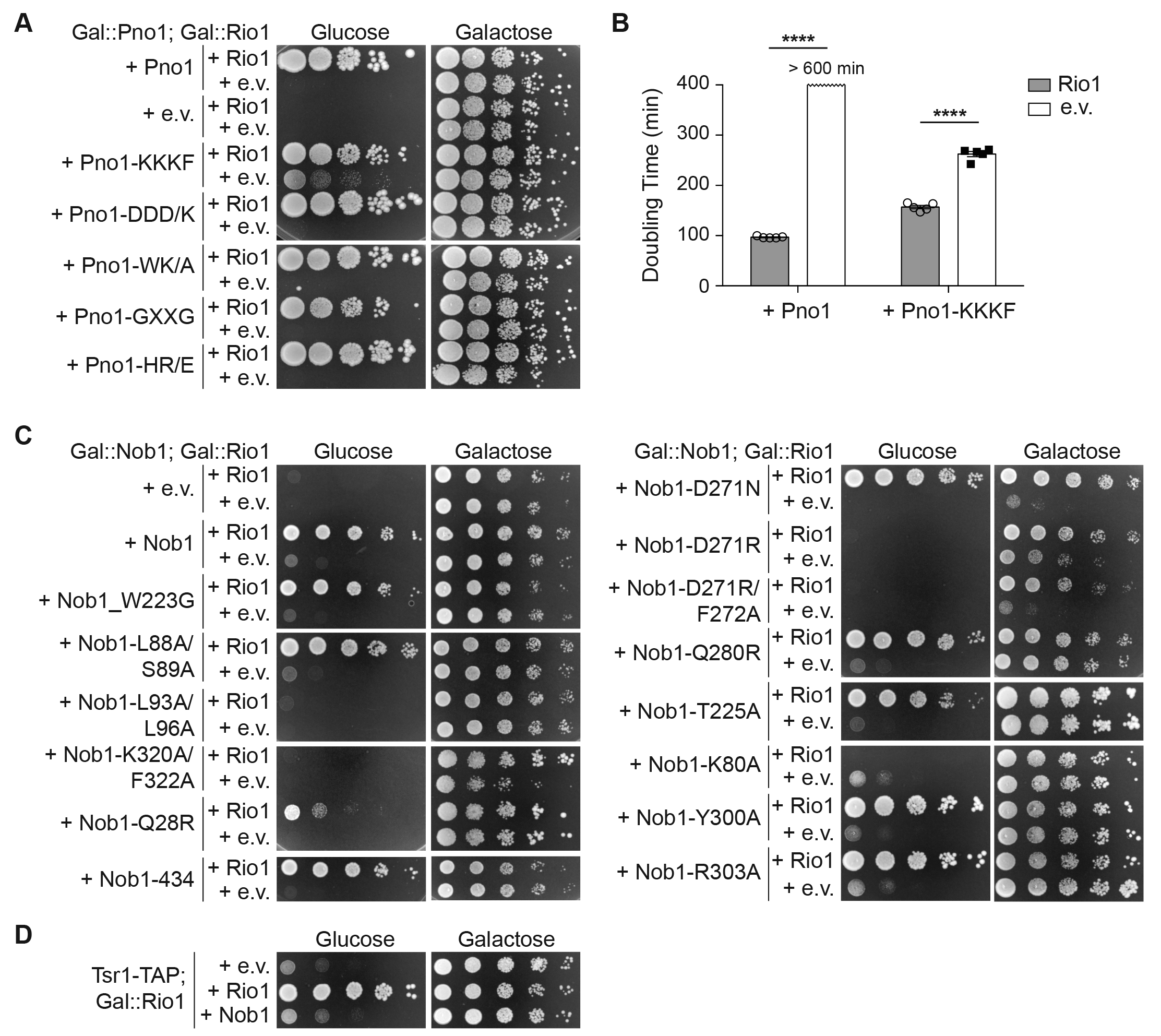

Supplement: S5 Fig — (A) Growth of cells expressing wild-type Pno1 or Pno1 mutants with and without Rio1 were compared by 10-fold serial dilutions on glucose and galactose dropout plates. Pno1-GXXG (N111G/S112K/W113D/T114G), Pno1-WK/A (W113A/K115A), Pno1-HR/E (H104E/R105E), Pno1-DDD/K (D167K/D169K/D170K). (B) Quantitative growth measurements for cells expressing Pno1 or Pno1-KKKF in the presence or absence of Rio1. Five biological replicates, error bars represent SEM, and ****p < 0.0001 via unpaired t test. Numerical data are listed in S1 Data. (C) Growth of cells expressing wild-type Nob1 or Nob1 mutants with or without Rio1 were compared by 10-fold serial dilutions on glucose and galactose dropout plates. (D) Growth of cells containing endogenous Rio1 under a Gal promoter expressing either wild-type Nob1 or Rio1 under a copper-inducible (Cup1) promoter or an empty vector were compared by 10-fold serial dilutions on glucose or galactose dropout plates with 100 μM CuSO4. Gal, galactose. (TIF) [file pbio.3000329.s005.tif]

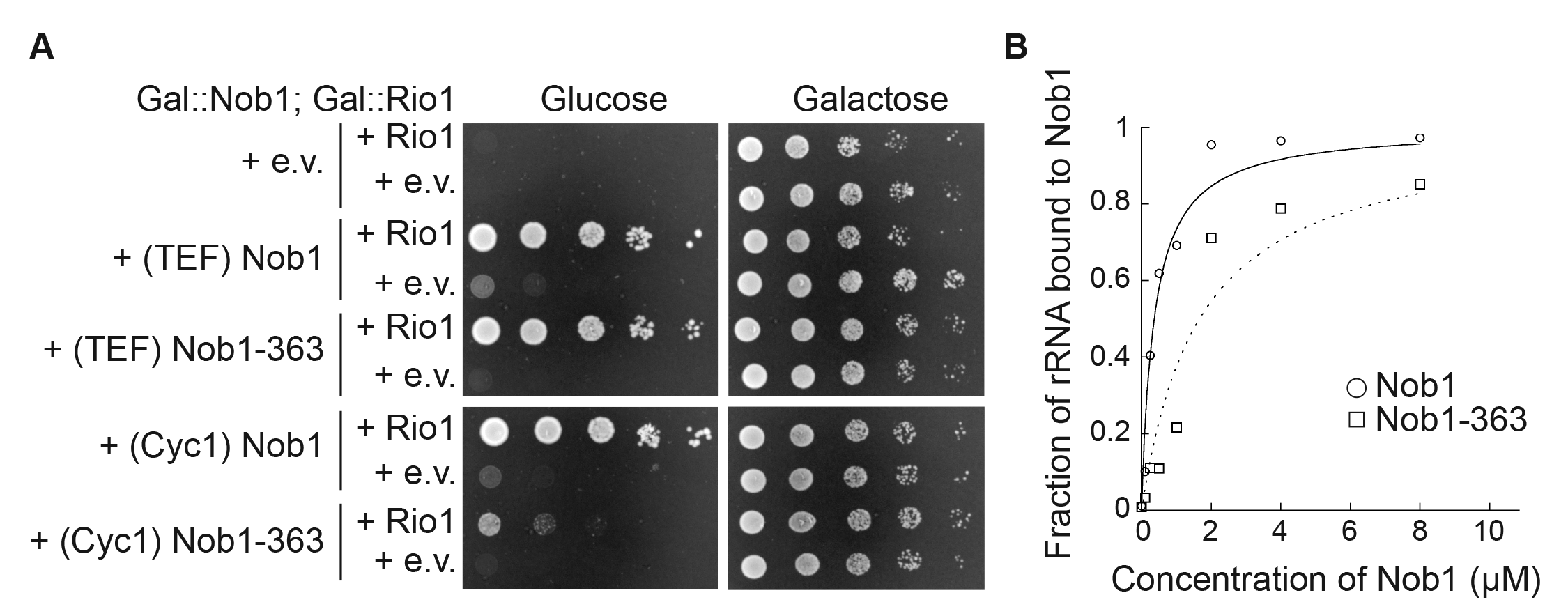

Supplement: S6 Fig — (A) Growth of cells expressing wild-type Nob1 or Nob1 mutants under the Tef2 or Cyc1 promoter, as indicated, with or without Rio1 were compared by 10-fold serial dilutions on glucose and galactose dropout plates. The Tef2 promoter produces higher protein levels [57]. (B) RNA binding assay with in vitro transcribed H44-A2 RNA (20S pre-rRNA mimic) and recombinant Nob1 or Nob1-363. Three independent experiments yielded values of Kd = 0.37 +/− 0.22 μM for Nob1 binding H44-A2 (white circles) and Kd = 1.43 +/− 0.22 μM for Nob1-363 binding H44-A2 (white squares). Numerical data are listed in S1 Data. (TIF) [file pbio.3000329.s006.tif]

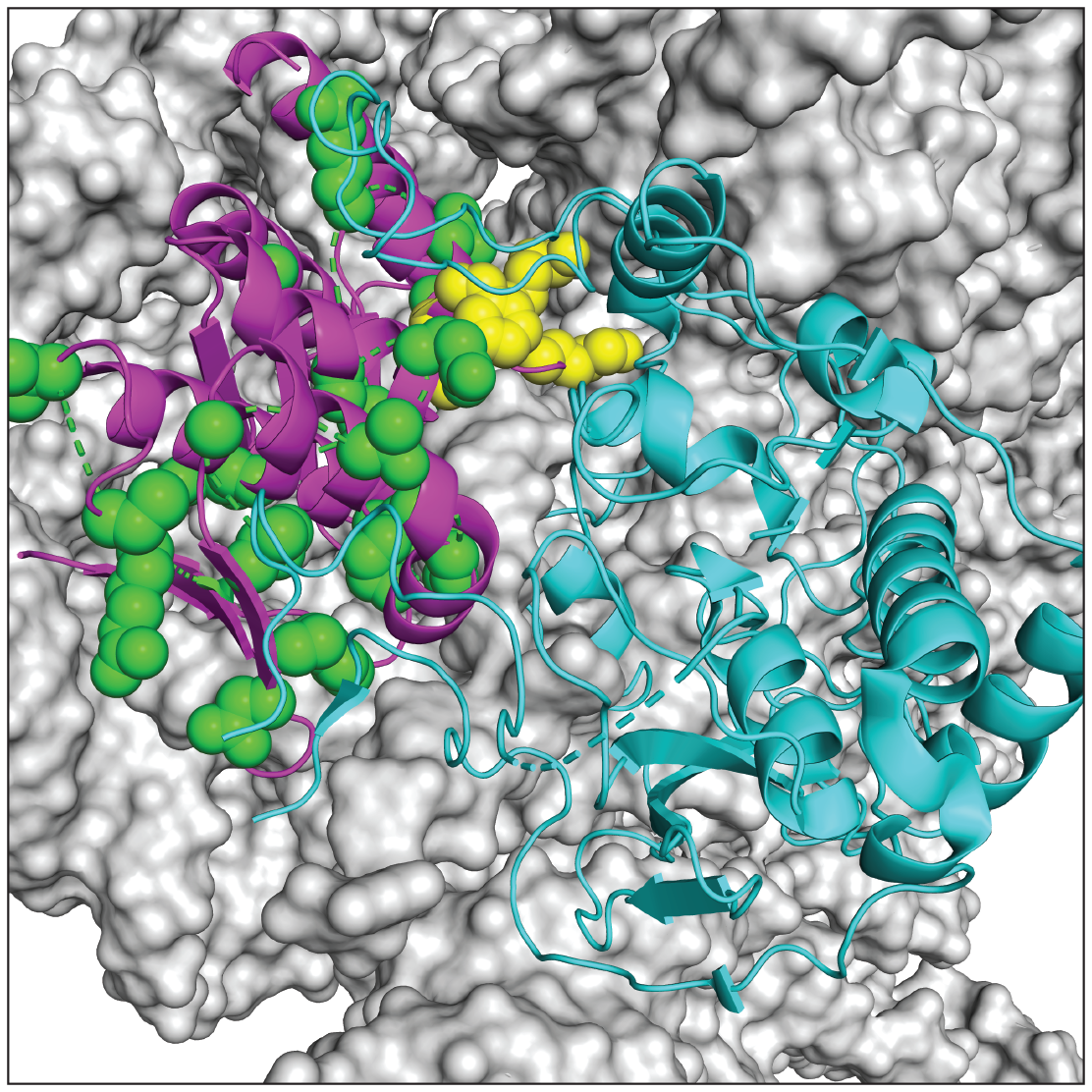

Supplement: S7 Fig — Mutations in Pno1 that accumulate in diverse cancers (green space fill, from the TCGA Research Network: https://www.cancer.gov/tcga) are directly adjacent to Pno1-KKKF (yellow space fill) or similarly contact either the rRNA, Nob1, or ribosomal proteins. Premature 18S rRNA (from human pre-40S, surface view in grey) bound by Nob1 (cyan) and Pno1 (magenta). Image was obtained from PDB 6G18 (human pre-40S state C, [22]). For simplicity, all proteins other than Nob1 and Pno1 are omitted. TCGA, The Cancer Genome Atlas. (TIF) [file pbio.3000329.s007.tif]
