# Supplementary material for: A kinase-dependent checkpoint prevents escape of immature ribosomes into the translating pool
Source: PLoS Biol. 2019 Dec 13;17(12):e3000329. doi: 10.1371/journal.pbio.3000329 (PMC6934326; doi:10.1371/journal.pbio.3000329)
Supplement: S1 Table — (DOCX) [file pbio.3000329.s008.docx]

**S1 Table**: Yeast strains used in this work

| **Strain** | **Description** | **Background** | **Genotype** | **Reference** |
| --- | --- | --- | --- | --- |
| YKK200 | WT | BY4741 | *MATα his3Δ1 leu2Δ0 met15Δ0 ura3Δ0* | GE Dharmacon |
| YKK650 | Gal::Nob1 | BY4741 | *MATα KanMX6::pGAL1-Nob1 his3Δ1 leu2Δ0 met15Δ0 ura3Δ0* | This work |
| YKK281 | Gal::Rio1  Tsr1-TAP | BY4741 | *MATα KanMX6::pGAL1-Rio1 his3::Tsr1-TAP leu2Δ0 met15Δ0 ura3Δ0* | This work |
| YKK1134 | Gal::Nob1  Gal::Rio1 | BY4741 | *MATα KanMX6::pGAL1-Nob1 NatMX6::pGal1-Rio1 his3Δ1 leu2Δ0 met15Δ0 ura3Δ0* | This work |
| YKK988 | Gal::Pno1  Gal::Rio1 | BY4741 | *MATα KanMX6::pGAL1-Pno1 NatMX6::pGAL1-Rio1 his3Δ1 leu2Δ0 met15Δ0 ura3Δ0* | This work |
| YKK1181 | Gal::Rio2 | BY4741 | *MATα KANMX6::pGAL1-Rio2 his3Δ1 leu2Δ0 met15Δ0 ura3Δ0* | This work |
| YKK218 | Gal::Fap7 | BY4741 | *MATα KANMX6::pGAL1-Fap7 his3Δ1 leu2Δ0 met15Δ0 ura3Δ0* | [5] |
| YKK438 | Gal::Hrr25 | BY4741 | *MATα NATMX6::pGAL1-Hrr25 his3Δ1 leu2Δ0 met15Δ0 ura3Δ0* | [58] |
