# Supplementary material for: A kinase-dependent checkpoint prevents escape of immature ribosomes into the translating pool
Source: PLoS Biol. 2019 Dec 13;17(12):e3000329. doi: 10.1371/journal.pbio.3000329 (PMC6934326; doi:10.1371/journal.pbio.3000329)
Supplement: S2 Table — (DOCX) [file pbio.3000329.s009.docx]

**S2 Table**: Plasmids used in this work

| **Plasmid** | **Description** | **Backbone** | **Reference** |
| --- | --- | --- | --- |
| pKK3464 | Gal::Nob1 | pRS426 | This work |
| pKK3723 | Gal::Nob1-D15N | pRS426 | This work |
| pKK30014 | CUP1::Nob1 | pRS426 | This work |
| pKK3387 | TEF::Nob1 | pRS413 | This work |
| pKK30177 | TEF::Nob1-W223G | pRS413 | This work |
| pKK378 | TEF::Nob1 | pRS416 | This work |
| pKK3241 | TEF::Nob1-D15N | pRS416 | This work |
| pKK30399 | TEF::Nob1-L88A/S89A | pRS416 | This work |
| pKK30400 | TEF::Nob1-L93A/L96A | pRS416 | This work |
| pKK3056 | TEF::Nob1-K320A/F322A | pRS416 | This work |
| pKK30401 | TEF::Nob1-D271N | pRS416 | This work |
| pKK30402 | TEF::Nob1-D271R | pRS416 | This work |
| pKK30403 | TEF::Nob1-D271R/F272A | pRS416 | This work |
| pKK30404 | TEF::Nob1-Q280R | pRS416 | This work |
| pKK3243 | TEF::Nob1-K80A | pRS416 | This work |
| pKK3246 | TEF::Nob1-R303A | pRS416 | This work |
| pKK3245 | TEF::Nob1-Y300A | pRS416 | This work |
| pKK3211 | TEF::Nob1-363 | pRS416 | This work |
| pKK377 | CYC1::Nob1 | pRS415 | This work |
| pKK396 | CYC1::Nob1-363 | pRS415 | This work |
| pKK397 | CYC1::Nob1-434 | pRS415 | This work |
| pKK381 | CYC1::Nob1-T225A | pRS415 | This work |
| pKK3124 | CYC1::Nob1-Q28R | pRS415 | This work |
| pKK30156 | TEF::Rio1 | pRS415 | This work |
| pKK3280 | TEF::Rio1 | pRS416 | This work |
| pKK3999 | CUP1::Rio1 | pRS426 | This work |
| pKK30028 | CUP1::Rio1 | pRS423 | This work |
| pKK30073 | CUP1::Rio1-D261A | pRS423 | This work |
| pKK30074 | CUP1::Rio1-K86A | pRS423 | This work |
| pKK3270 | TEF::Pno1 | pRS416 | [18] |
| pKK3823 | TEF::Pno1-KKKF | pRS416 | [23] |
| pKK3271 | TEF::Pno1-GxxG | pRS416 | [18] |
| pKK3272 | TEF::Pno1-WK/A | pRS416 | This work |
| pKK3273 | TEF::Pno1-HR/E | pRS416 | [18] |
| pKK3274 | TEF::Pno1-DDD/K | pRS416 | [18] |
| pKK30157 | TEF::TAP-Pno1 | pRS413 | This work |
| pKK30059 | ADH1/GPD-Start-site (Renilla(AUG)/Firefly(UUG)) | pRS415 | [41] |
| pKK3920 | ADH1-0 Frameshift Control | pRS415 | [38] |
| pKK3921 | ADH1-liter_A_ (-1) Frameshift | pRS415 | [38] |
| pKK3922 | ADH1-Ty1 (+1) Frameshift | pRS415 | [38] |
| pKK3923 | TEF-Read-through | pRS415 | [39] |
| pKK3924 | TEF-Stop | pRS415 | [39] |
| pKK3925 | TEF-Misincorporation (Firefly_H245R) | pRS415 | [40] |
| pKK1181 | MBP-Rio1 | pSV272 | This work |
| pKK187 | MBP-Nob1 | pSV272 | [16] |
| pKK1051 | MBP-Nob1-363 | pSV272 | This work |
| pKK191 | MBP-Pno1 | pSV272 | [18] |
| pKK3819 | TEF::Rio2 | pRS415 | This work |
| pKK3001 | Cyc1::Fap7 | pRS415 | [6] |
| pKK30038 | TEF::Hrr25 | pRS415 | This work |
