# Supplementary material for: A kinase-dependent checkpoint prevents escape of immature ribosomes into the translating pool
Source: PLoS Biol. 2019 Dec 13;17(12):e3000329. doi: 10.1371/journal.pbio.3000329 (PMC6934326; doi:10.1371/journal.pbio.3000329)

1A Gal::Nob1

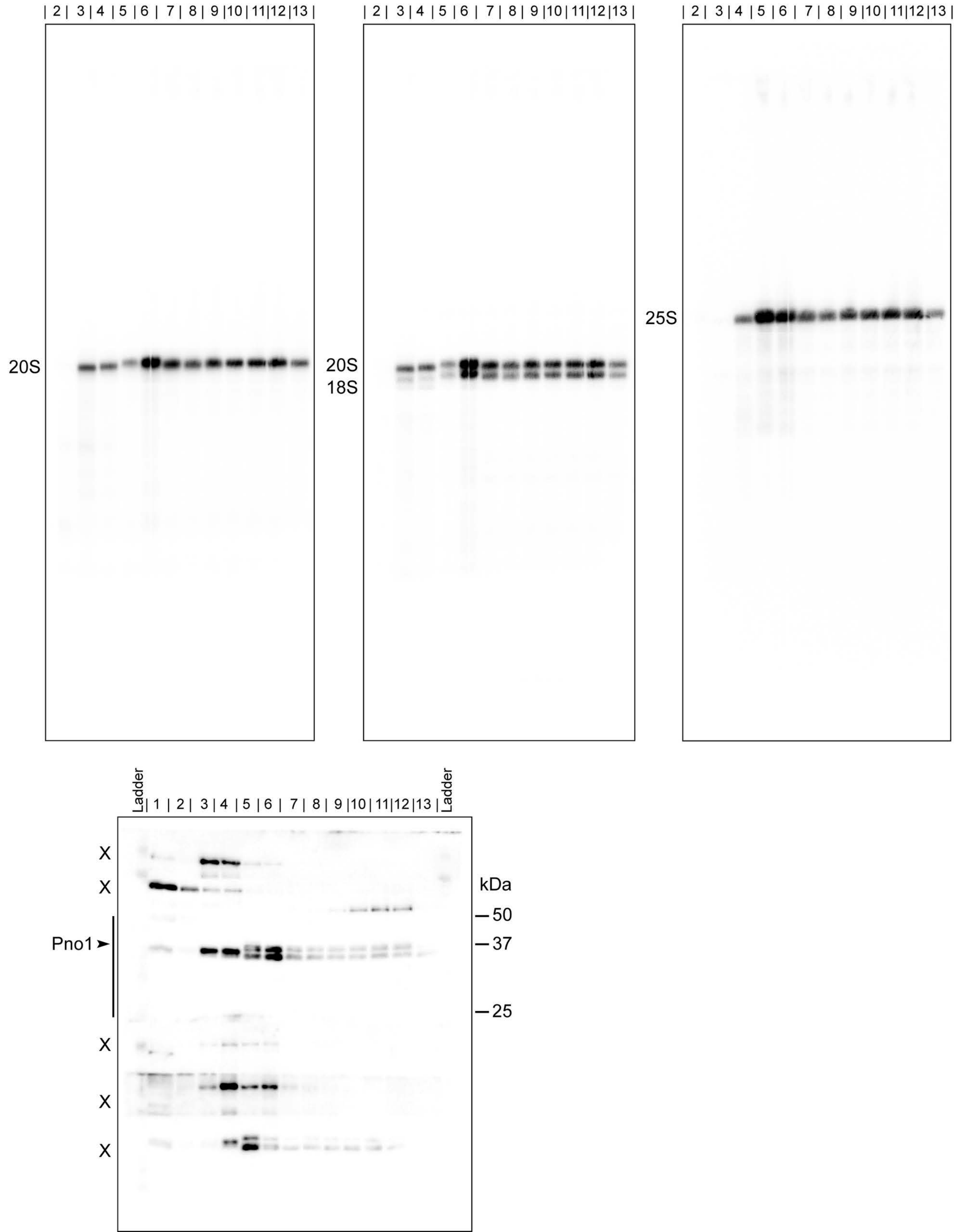

| 2 | 3 | 4 | 5 | 6 | 7 | 8 | 9 | 10 | 11 | 12 | 13 |
20S
| 2 | 3 | 4 | 5 | 6 | 7 | 8 | 9 | 10 | 11 | 12 | 13 |
20S
18S
| 2 | 3 | 4 | 5 | 6 | 7 | 8 | 9 | 10 | 11 | 12 | 13 |
25S
Ladder | 1 | 2 | 3 | 4 | 5 | 6 | 7 | 8 | 9 | 10 | 11 | 12 | 13 | Ladder
X
X
Pno1
X
X
X
kDa
50
37
25

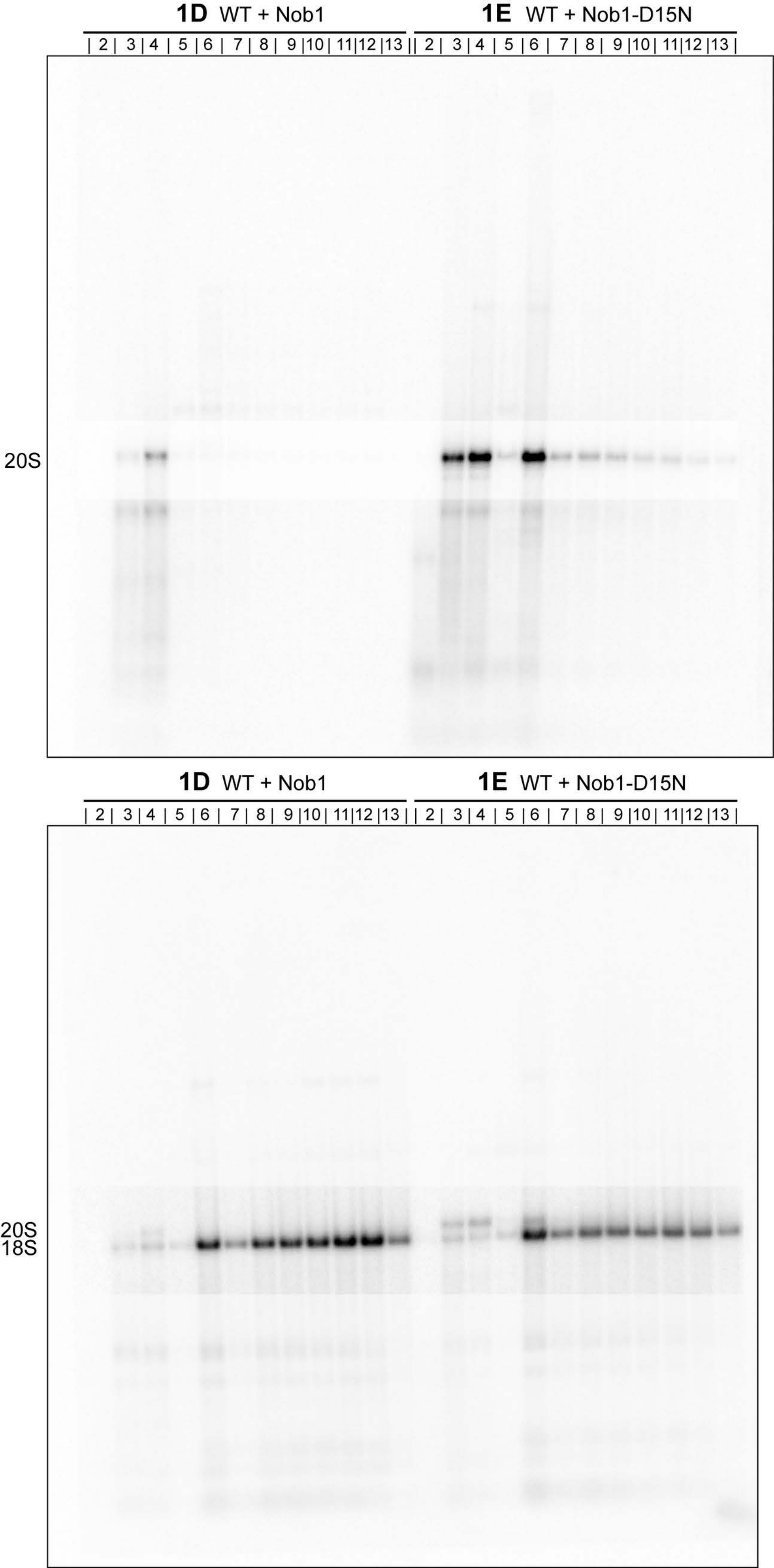
1D WT + Nob1
1E WT + Nob1-D15N
| 2 | 3 | 4 | 5 | 6 | 7 | 8 | 9 | 10 | 11 | 12 | 13 |
| 2 | 3 | 4 | 5 | 6 | 7 | 8 | 9 | 10 | 11 | 12 | 13 |
20S
1D WT + Nob1
1E WT + Nob1-D15N
| 2 | 3 | 4 | 5 | 6 | 7 | 8 | 9 | 10 | 11 | 12 | 13 |
| 2 | 3 | 4 | 5 | 6 | 7 | 8 | 9 | 10 | 11 | 12 | 13 |
20S
18S

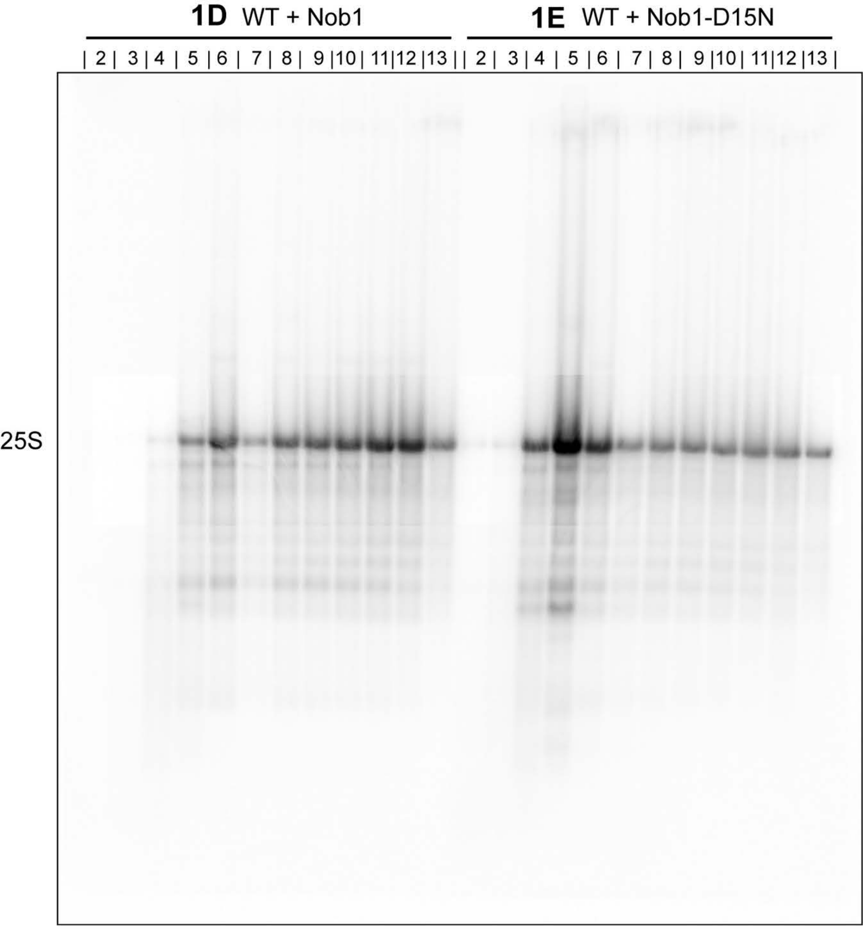
1D WT + Nob1
1E WT + Nob1-D15N
2 3 4 5 6 7 8 9 10 11 12 13
2 3 4 5 6 7 8 9 10 11 12 13
25S

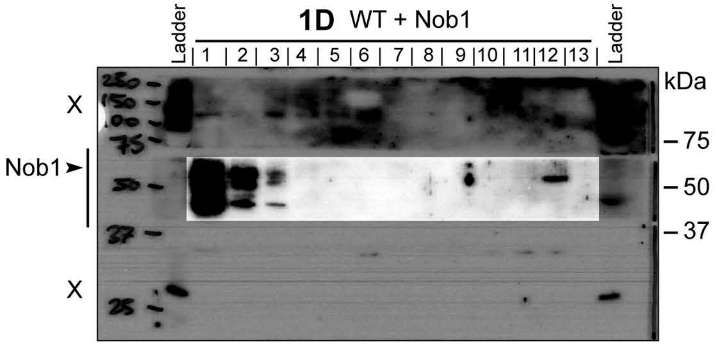
1D WT + Nob1
Ladder
1 2 3 4 5 6 7 8 9 10 11 12 13
Ladder
kDa
75
50
37
X
Nob1
X

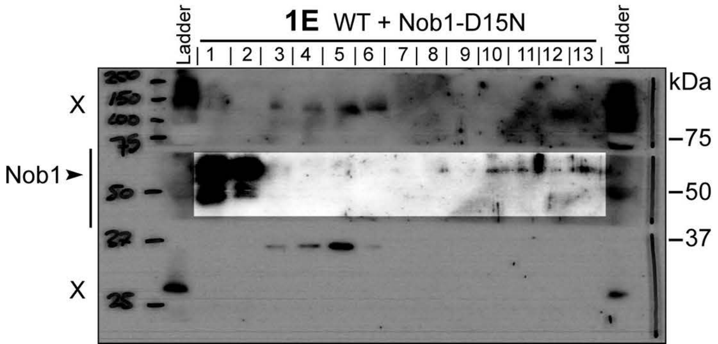
1E WT + Nob1-D15N
Ladder
1 2 3 4 5 6 7 8 9 10 11 12 13
Ladder
kDa
75
50
37
X
Nob1
X

1F WT + TEF::Nob1

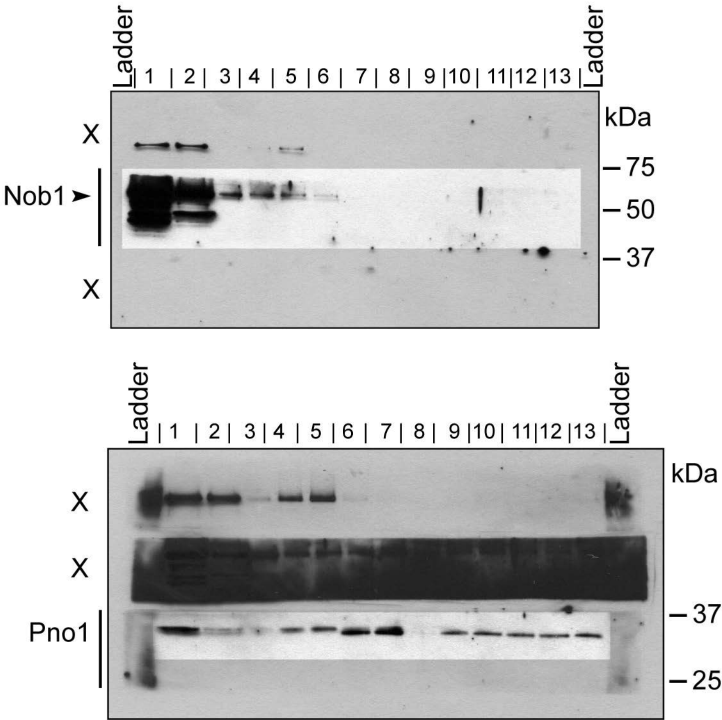

3B

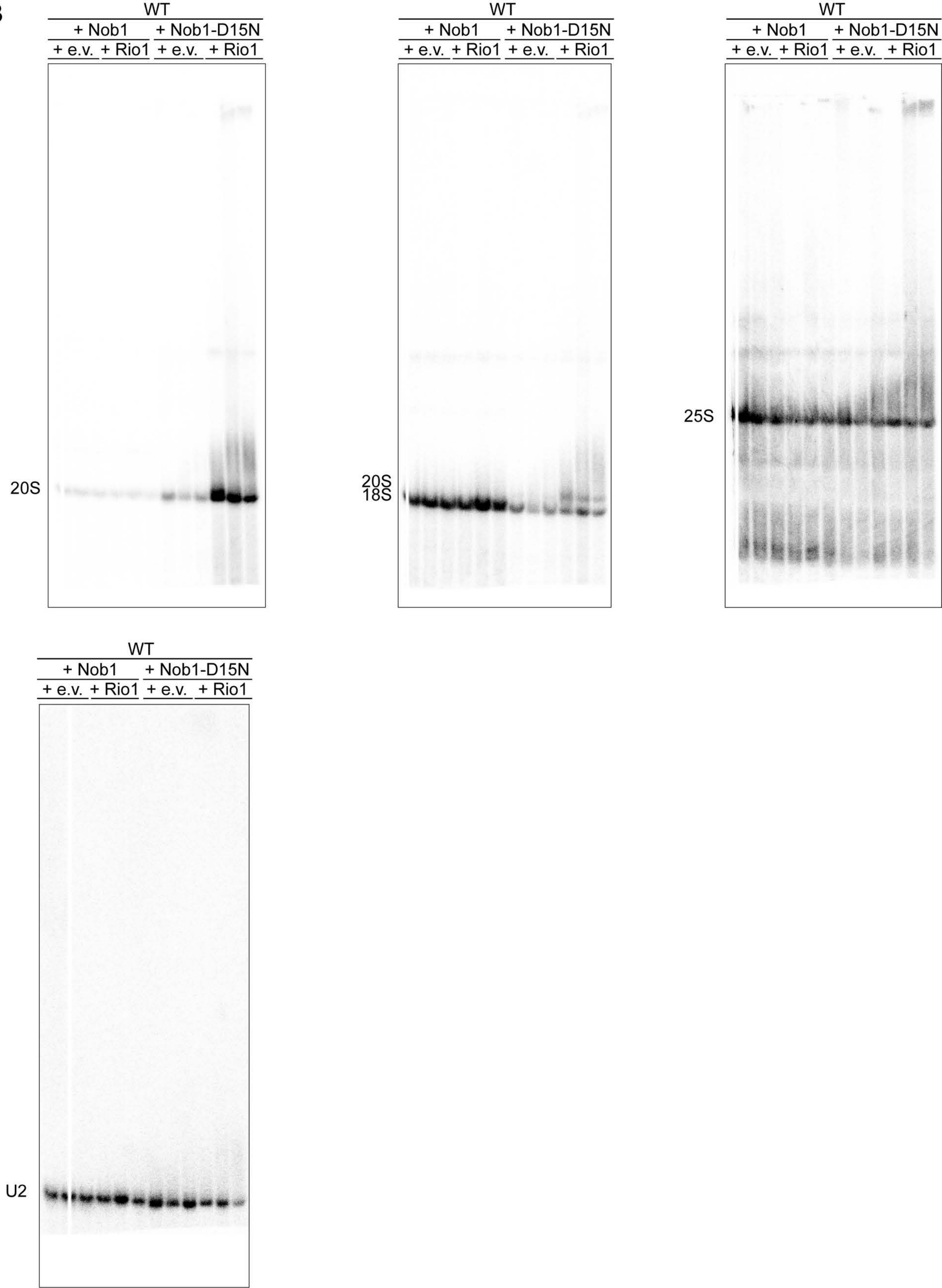

WT
+ Nob1
+ Nob1-D15N
+ e.v.
+ Rio1
+ e.v.
+ Rio1
20S
WT
+ Nob1
+ Nob1-D15N
+ e.v.
+ Rio1
+ e.v.
+ Rio1
20S
18S
WT
+ Nob1
+ Nob1-D15N
+ e.v.
+ Rio1
+ e.v.
+ Rio1
25S
WT
+ Nob1
+ Nob1-D15N
+ e.v.
+ Rio1
+ e.v.
+ Rio1
U2

3C

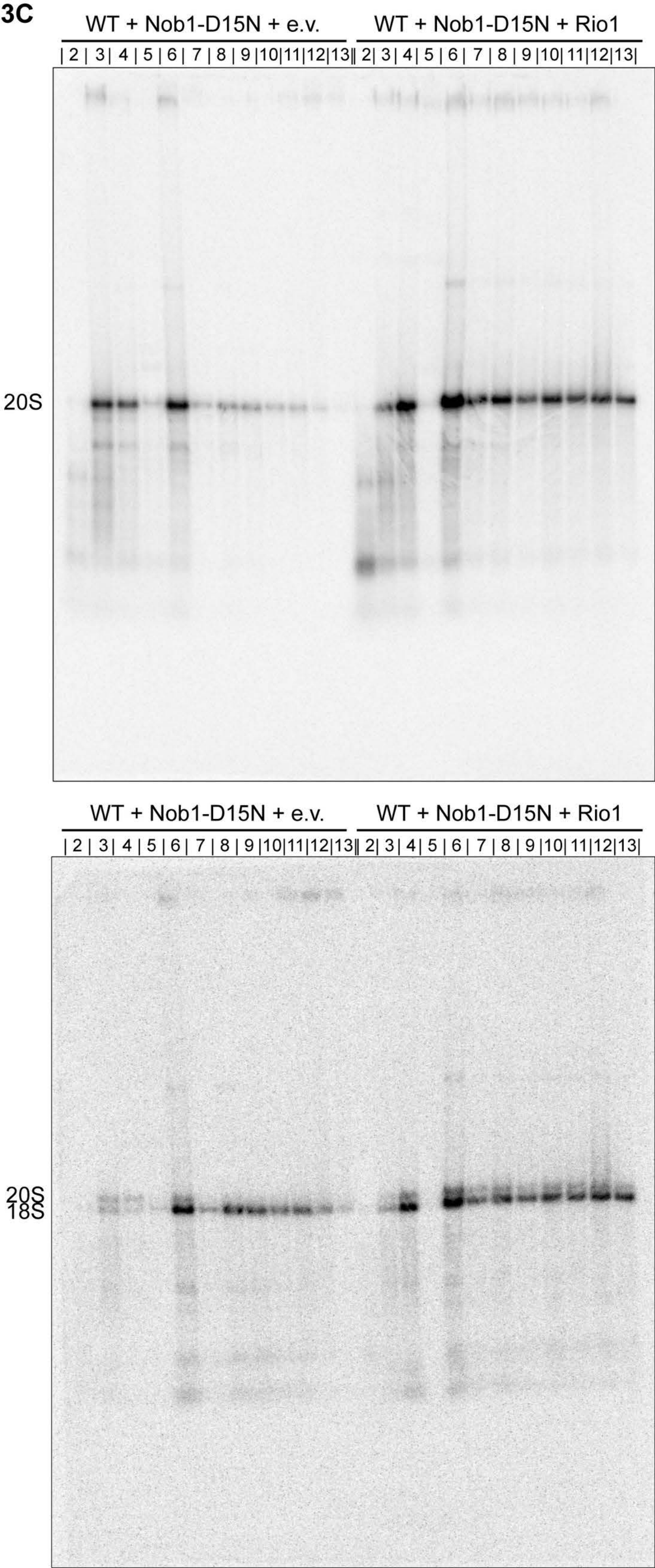
WT + Nob1-D15N + e.v.
WT + Nob1-D15N + Rio1
2 3 4 5 6 7 8 9 10 11 12 13
2 3 4 5 6 7 8 9 10 11 12 13
20S
WT + Nob1-D15N + e.v.
WT + Nob1-D15N + Rio1
2 3 4 5 6 7 8 9 10 11 12 13
2 3 4 5 6 7 8 9 10 11 12 13
20S
18S

3C

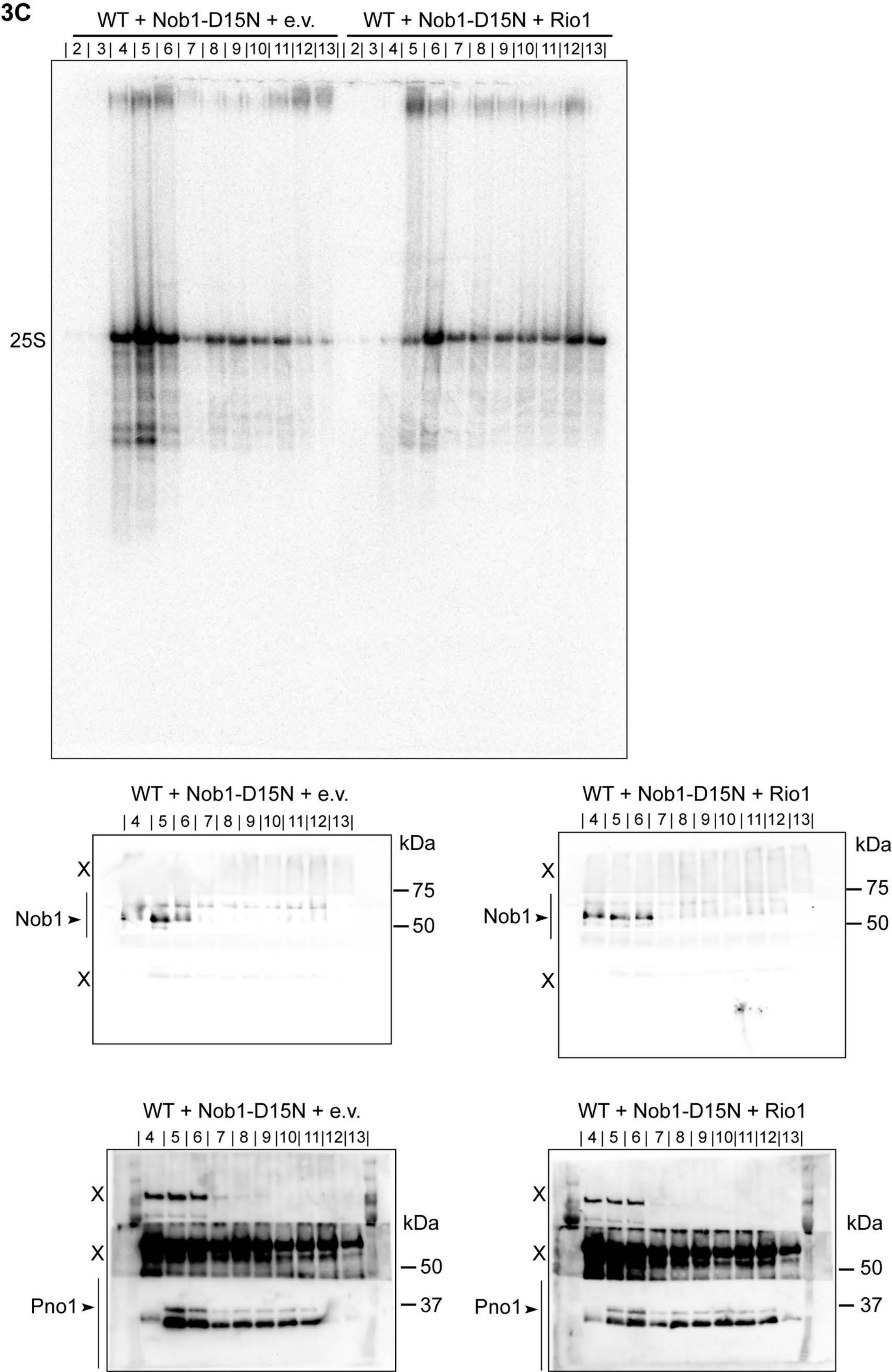

WT + Nob1-D15N + e.v.
WT + Nob1-D15N + Rio1
| 2 | 3 | 4 | 5 | 6 | 7 | 8 | 9 |10| 11|12|13|
| 2| 3 | 4 | 5 | 6 | 7 | 8 | 9 |10| 11|12|13|
25S
WT + Nob1-D15N + e.v.
| 4 | 5 | 6 | 7 | 8 | 9 |10| 11|12|13|
kDa
X
75
Nob1
50
X
WT + Nob1-D15N + Rio1
| 4 | 5 | 6 | 7 | 8 | 9 |10| 11|12|13|
kDa
X
75
Nob1
50
X
WT + Nob1-D15N + e.v.
| 4 | 5 | 6 | 7 | 8 | 9 |10| 11|12|13|
X
kDa
X
50
Pno1
37
WT + Nob1-D15N + Rio1
| 4 | 5 | 6 | 7 | 8 | 9 |10|11|12|13|
X
kDa
X
50
Pno1
37

4A

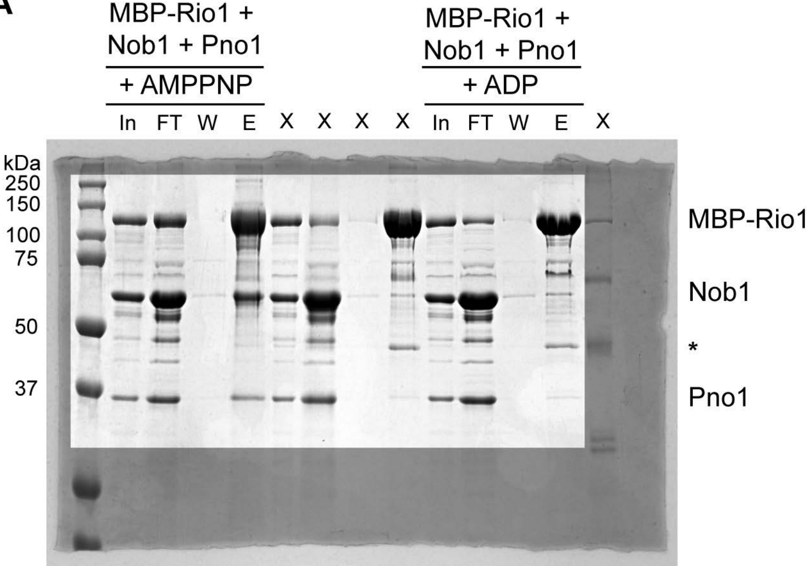

MBP-Rio1 + Nob1 + Pno1
+ AMPPNP
MBP-Rio1 + Nob1 + Pno1
+ ADP
In FT W E X X X X In FT W E X
kDa
250
150
100
75
50
37
MBP-Rio1
Nob1
*
Pno1

4B

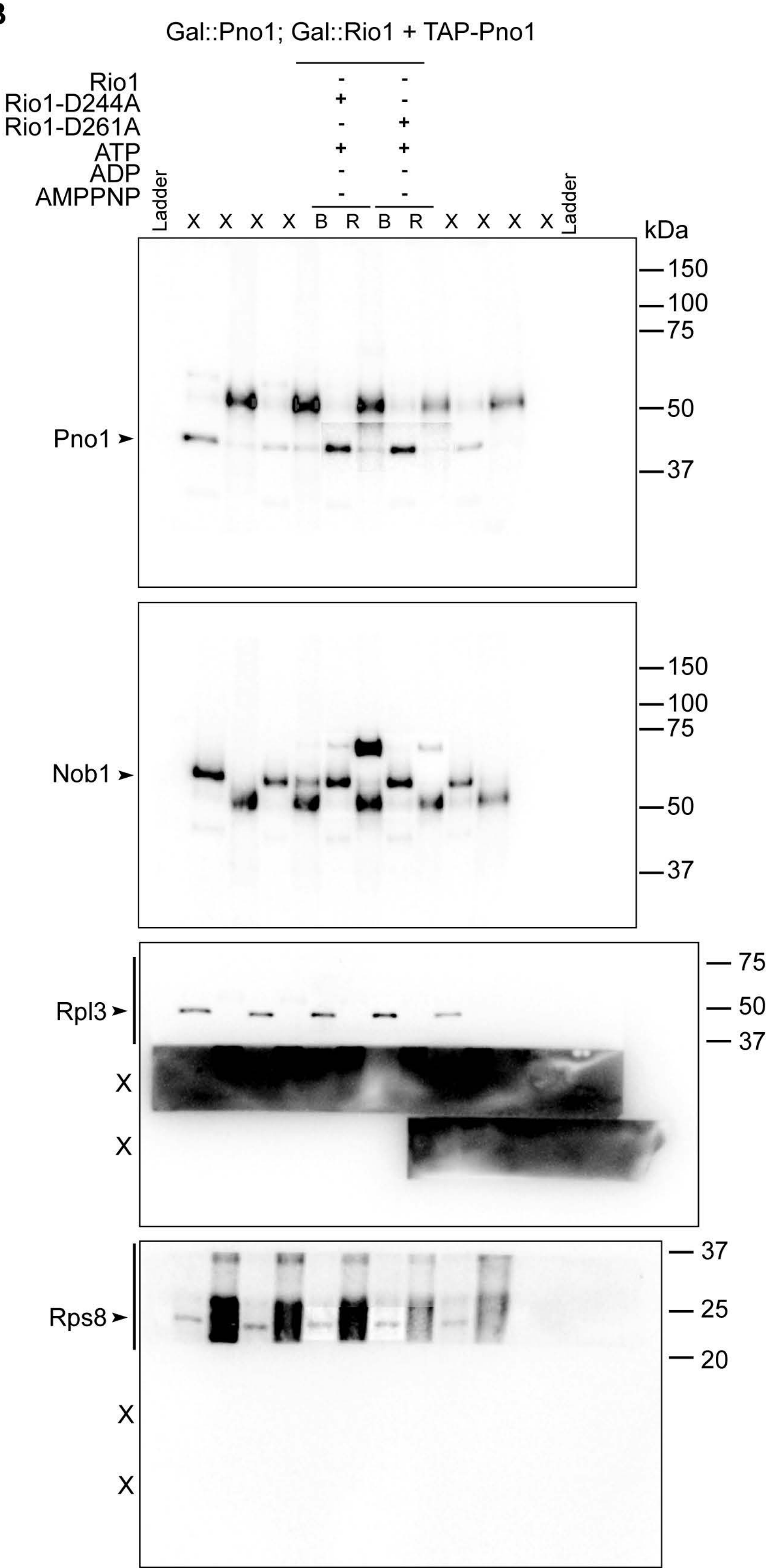

Gal::Pno1; Gal::Rio1 + TAP-Pno1
Rio1
Rio1-D244A
Rio1-D261A
ATP
ADP
AMPPNP
Ladder
X X X X B R B R X X X X
Ladder
kDa
150
100
75
50
37
Pno1
Nob1
Rpl3
Rps8
X
25
20

4B

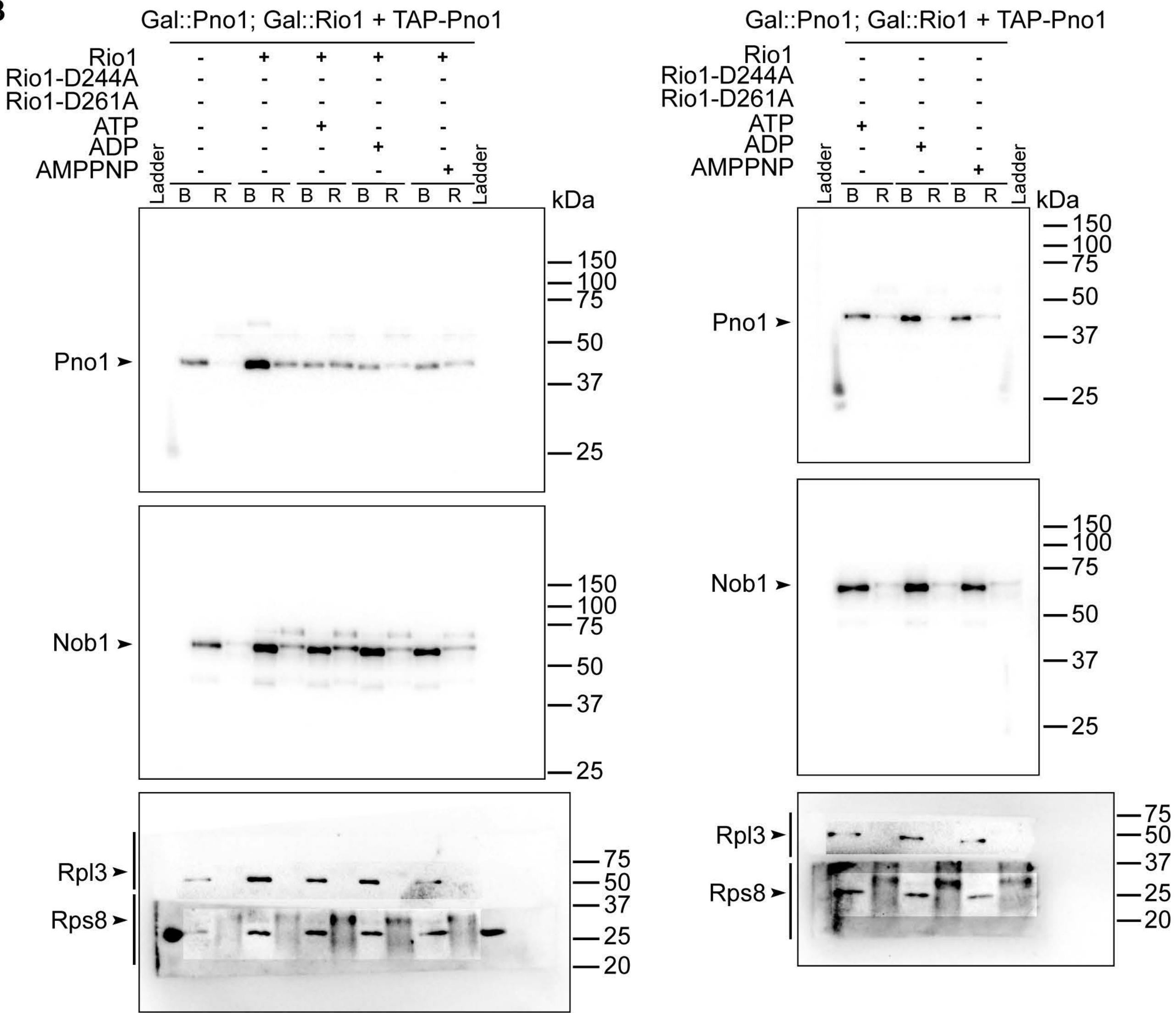

Gal::Pno1; Gal::Rio1 + TAP-Pno1
Rio1
Rio1-D244A
Rio1-D261A
ATP
ADP
AMPPNP
Ladder
B R B R B R B R B R
kDa
Pno1
Nob1
Rpl3
Rps8
150
100
75
50
37
25
20
Gal::Pno1; Gal::Rio1 + TAP-Pno1
Rio1
Rio1-D244A
Rio1-D261A
ATP
ADP
AMPPNP
Ladder
B R B R B R
kDa
Pno1
Nob1
Rpl3
Rps8

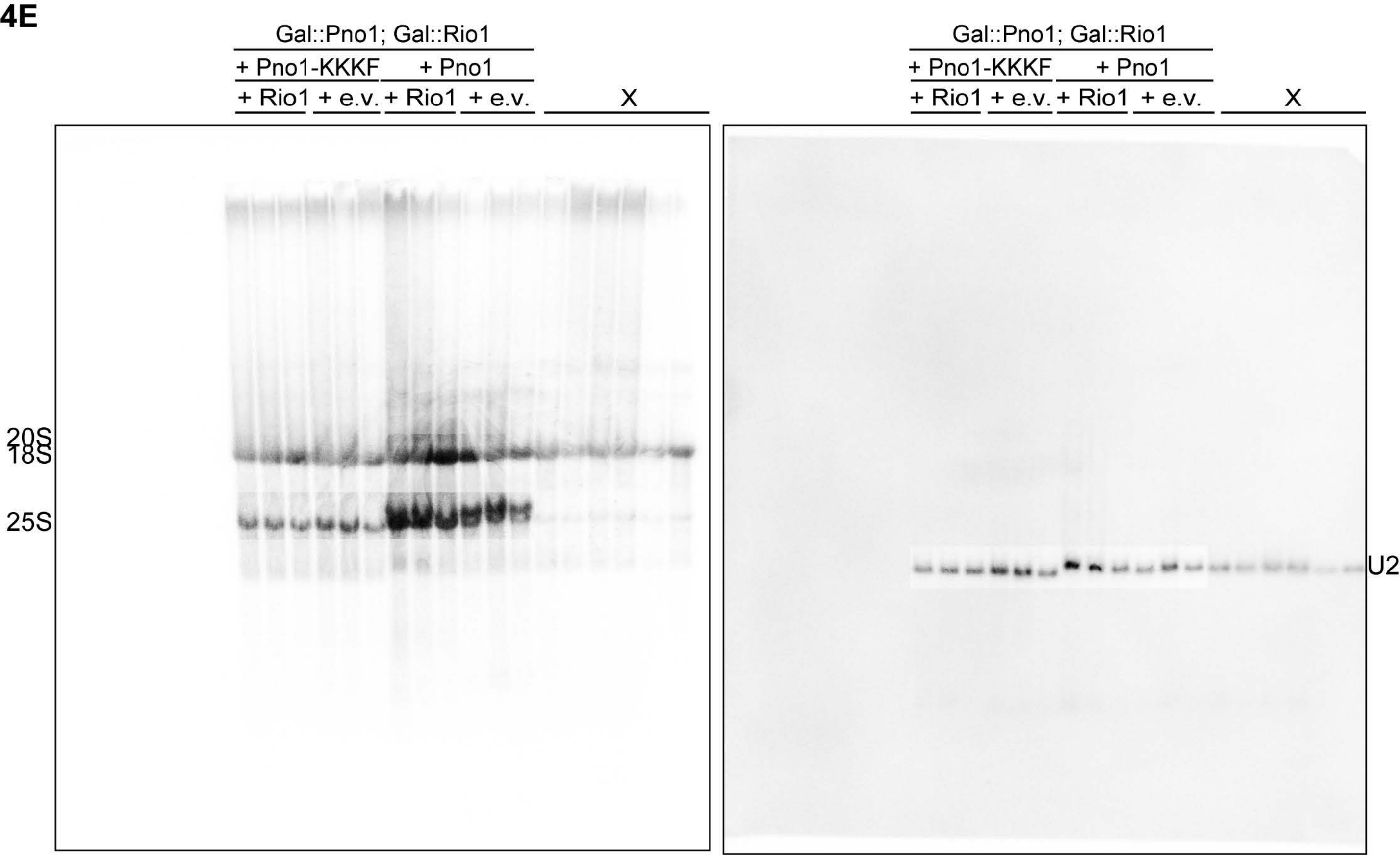
4E
Gal::Pno1; Gal::Rio1
+ Pno1-KKKF
+ Pno1
+ Rio1
+ e.v.
+ Rio1
+ e.v.
X
20S
18S
25S
Gal::Pno1; Gal::Rio1
+ Pno1-KKKF
+ Pno1
+ Rio1
+ e.v.
+ Rio1
+ e.v.
X
U2

4E

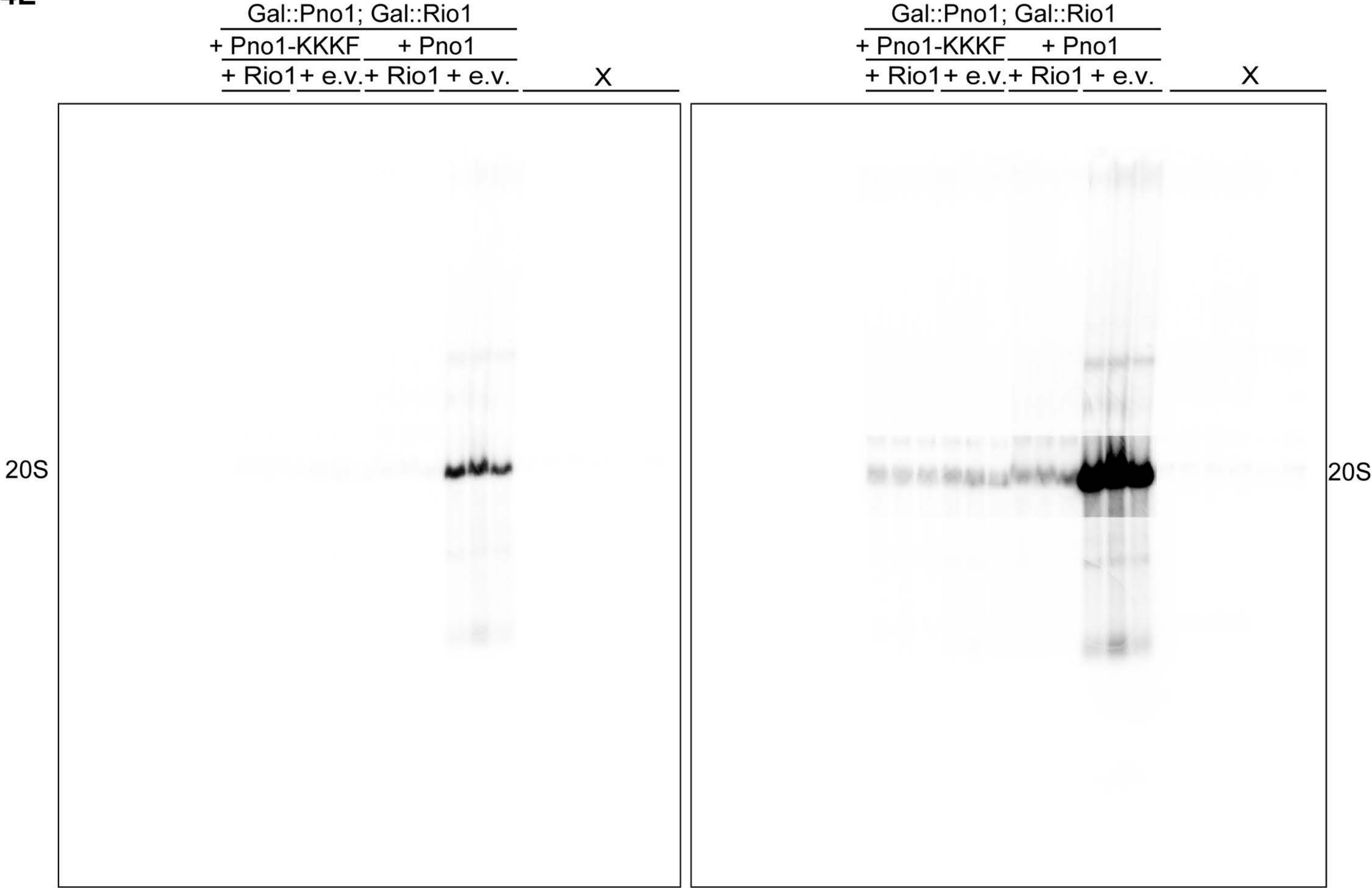
Gal::Pno1; Gal::Rio1
+ Pno1-KKKF
+ Pno1
+ Rio1
+ e.v.
+ Rio1
+ e.v.
X
20S
Gal::Pno1; Gal::Rio1
+ Pno1-KKKF
+ Pno1
+ Rio1
+ e.v.
+ Rio1
+ e.v.
X
20S

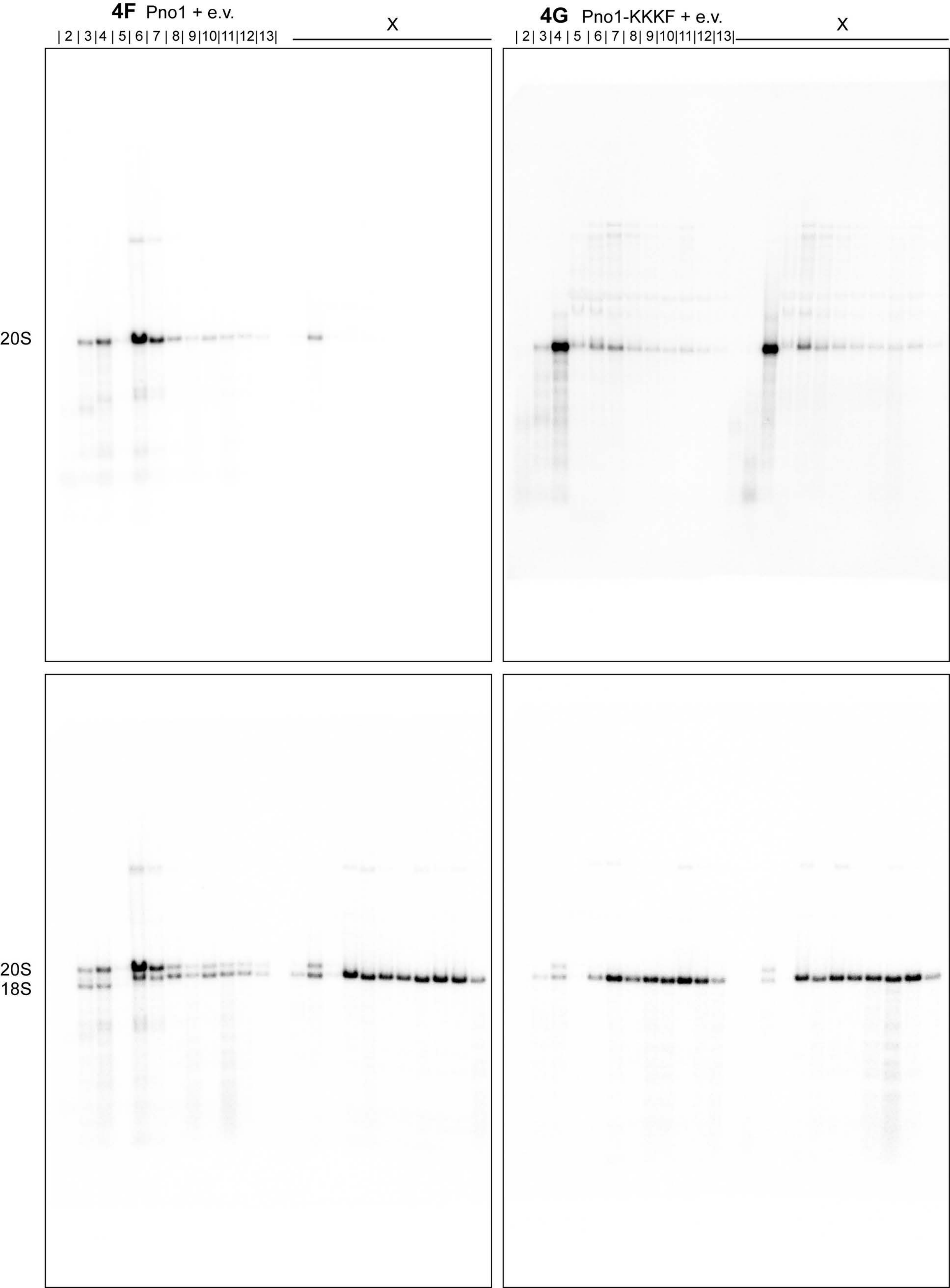

4F Pno1 + e.v.
| 2 | 3 | 4 | 5 | 6 | 7 | 8 | 9 | 10 | 11 | 12 | 13 |
X
4G Pno1-KKKF + e.v.
| 2 | 3 | 4 | 5 | 6 | 7 | 8 | 9 | 10 | 11 | 12 | 13 |
X
20S
20S
18S

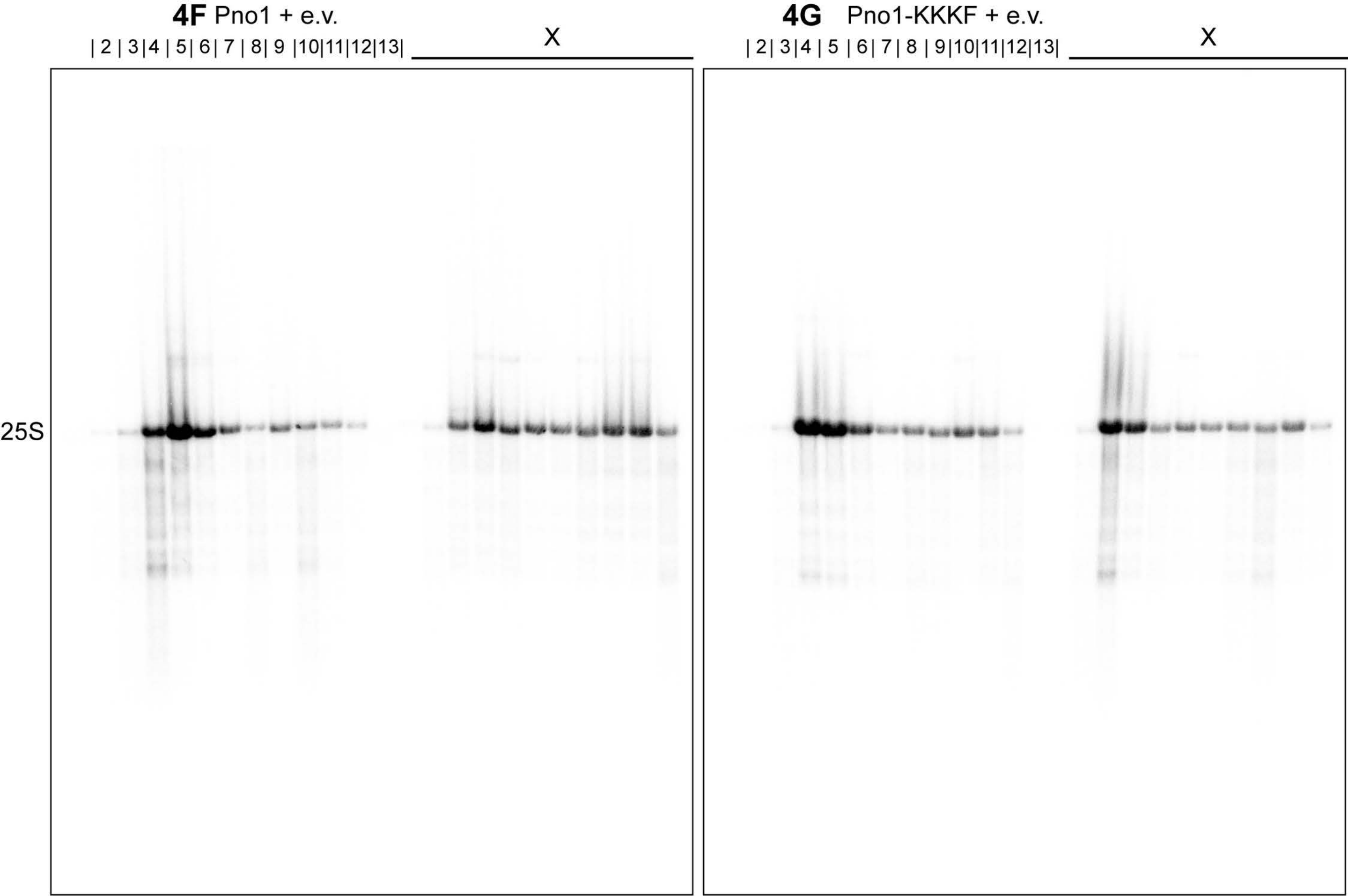

4F Pno1 + e.v.
| 2 | 3 |4 | 5 | 6| 7 | 8| 9 |10|11|12|13|
X
4G Pno1-KKKF + e.v.
| 2| 3 |4 | 5 | 6| 7 | 8 | 9 |10|11|12|13|
X
25S

5A

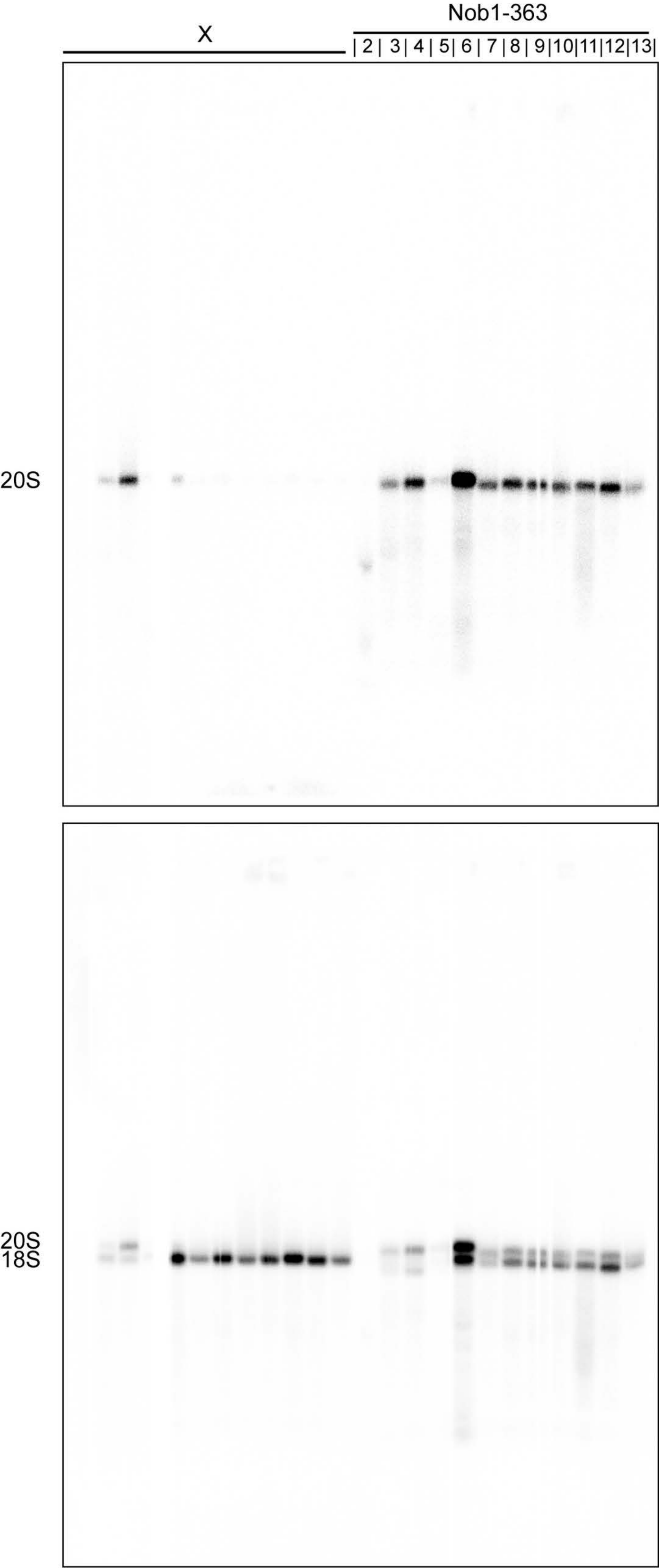

5A

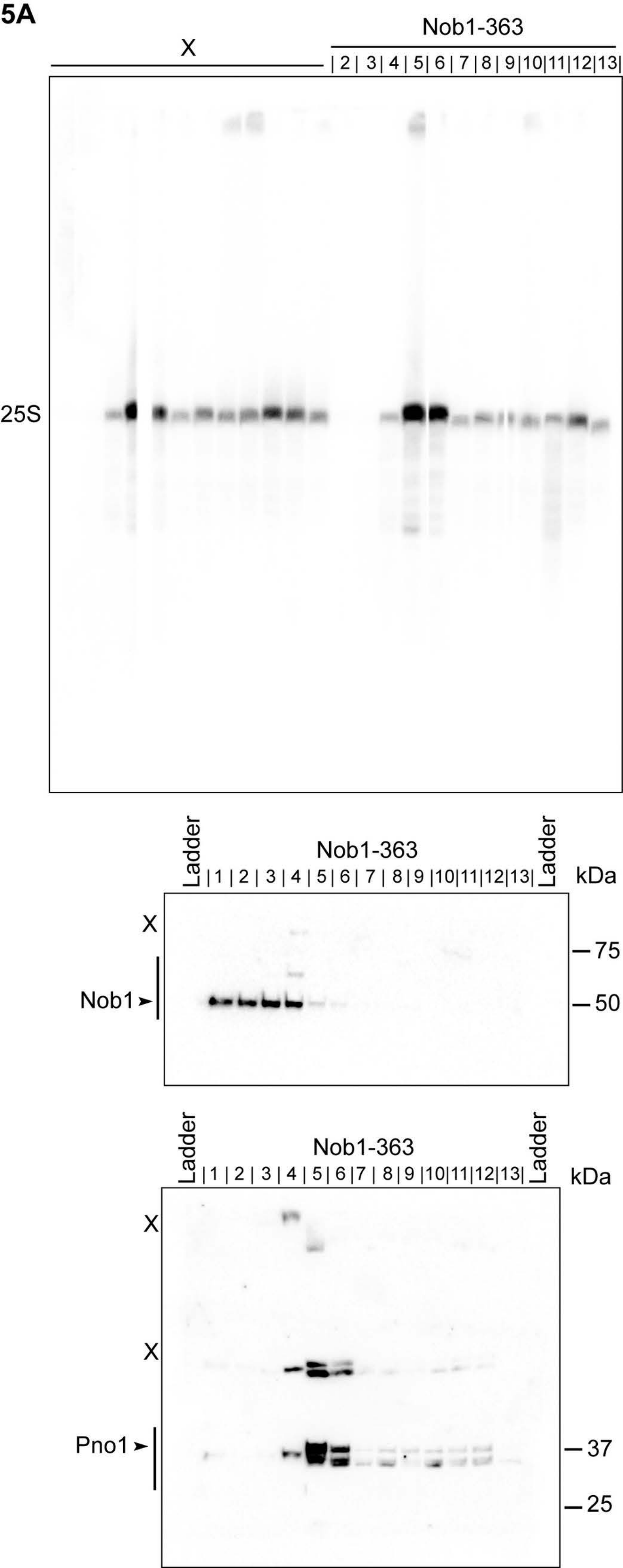
X
Nob1-363
| 2 | 3 | 4 | 5 | 6 | 7 | 8 | 9 |10 |11 |12 |13 |
25S
Ladder
Nob1-363
| 1 | 2 | 3 | 4 | 5 | 6 | 7 | 8 | 9 |10 |11 |12 |13 |
Ladder
kDa
X
Nob1
75
50
Ladder
Nob1-363
| 1 | 2 | 3 | 4 | 5 | 6 | 7 | 8 | 9 |10 |11 |12 |13 |
Ladder
kDa
X
X
Pno1
37
25

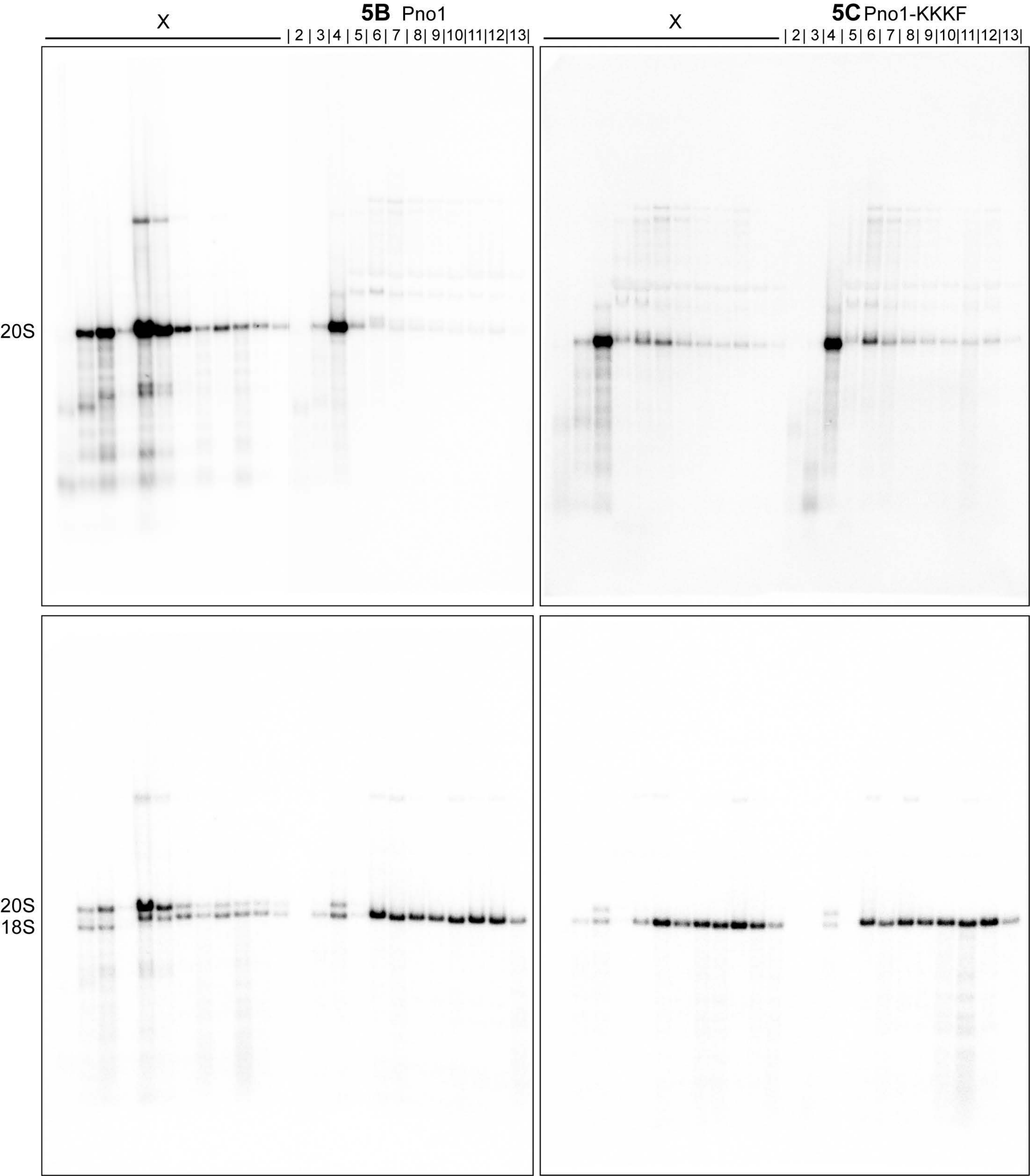
X
5B Pno1
| 2 | 3 | 4 | 5 | 6 | 7 | 8 | 9 |10|11|12|13|
X
5C Pno1-KKKF
| 2 | 3 | 4 | 5 | 6 | 7 | 8 | 9 |10|11|12|13|
20S
20S
18S

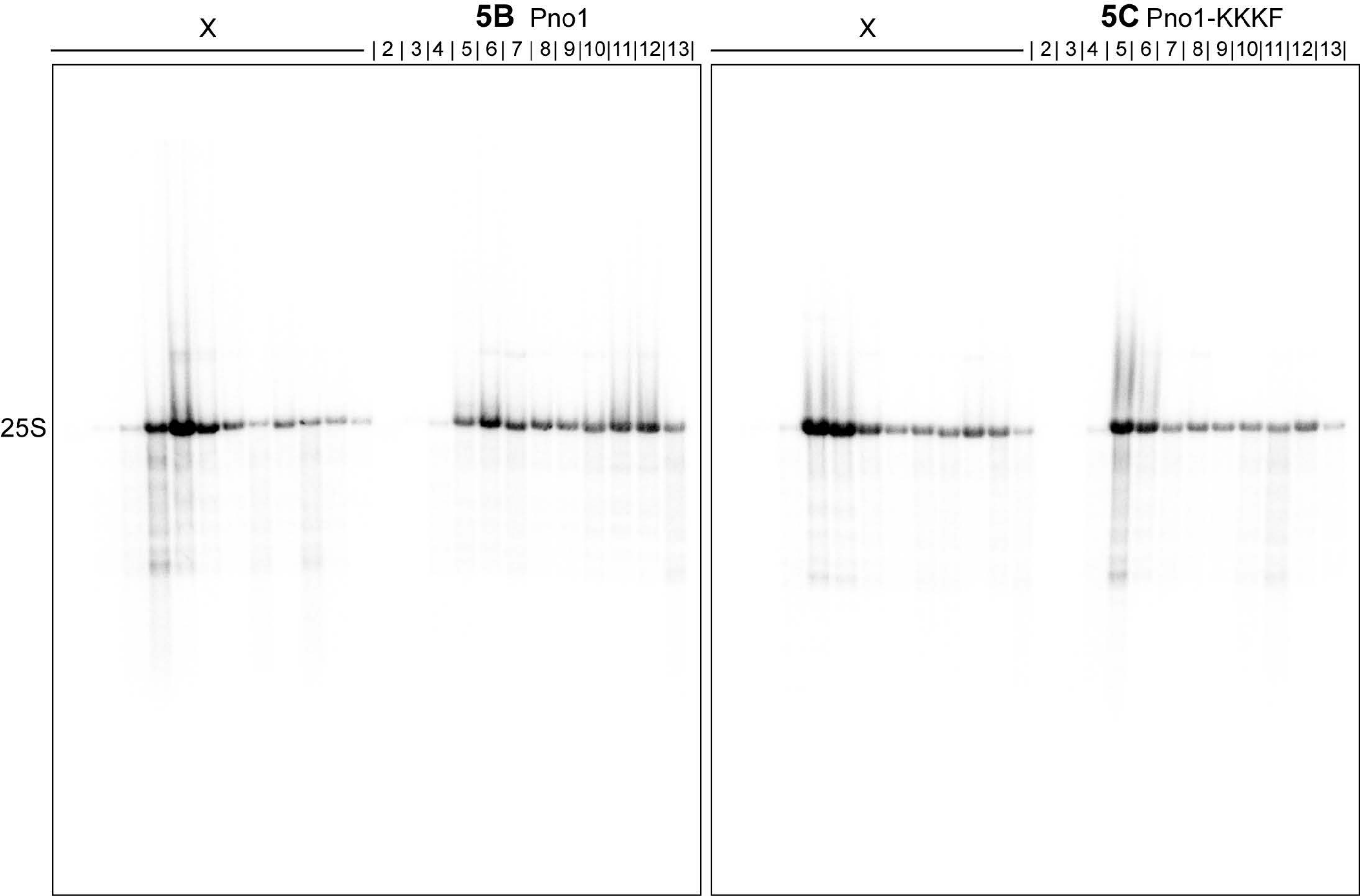
5B Pno1
X
| 2 | 3 |4 | 5| 6| 7 | 8| 9|10|11|12|13|
5C Pno1-KKKF
X
| 2| 3 |4 | 5| 6| 7| 8| 9|10|11|12|13|
25S

S1A

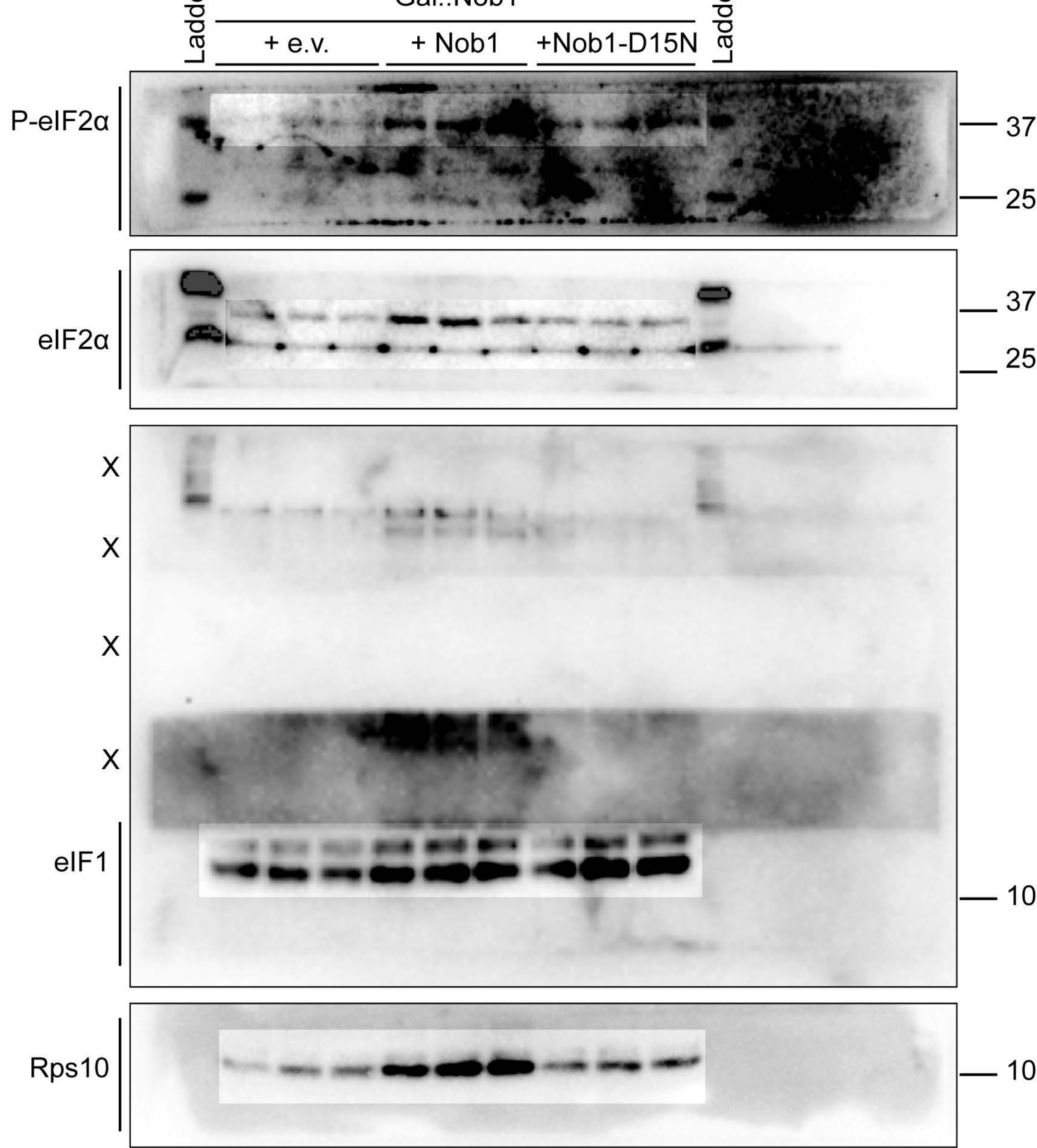
Ladder
Gal::Nob1
+ e.v.
+ Nob1
+Nob1-D15N
Ladder
P-eIF2α
37
25
eIF2α
37
25
X
X
X
X
eIF1
10
Rps10
10

S1C

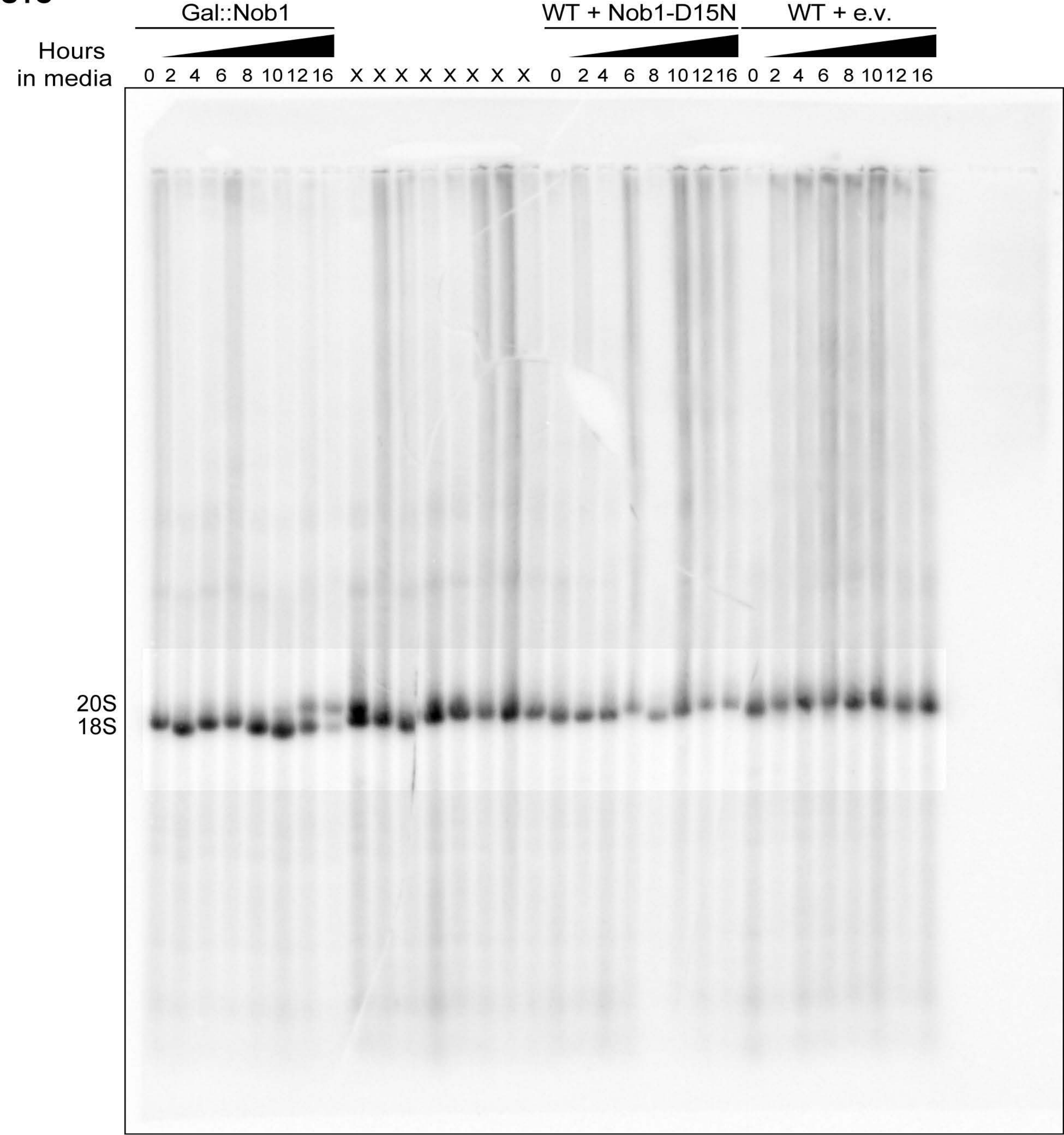
Gal::Nob1
WT + Nob1-D15N
WT + e.v.
Hours in media
0 2 4 6 8 10 12 16 X X X X X X X X 0 2 4 6 8 10 12 16 0 2 4 6 8 10 12 16
20S
18S

S1C

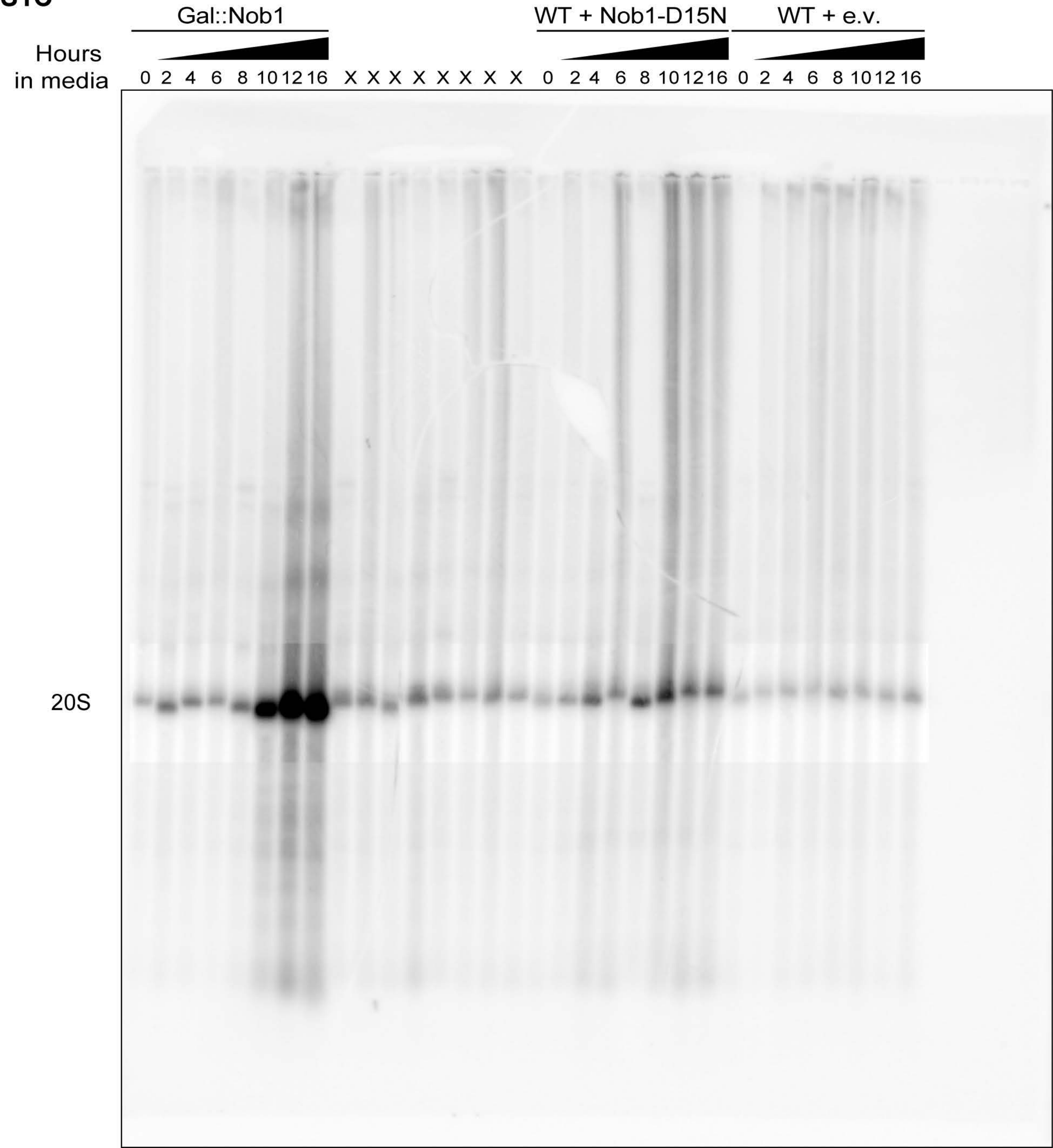
Gal::Nob1
WT + Nob1-D15N
WT + e.v.
Hours
in media
0 2 4 6 8 10 12 16 X X X X X X X X 0 2 4 6 8 10 12 16 0 2 4 6 8 10 12 16
20S

S1C

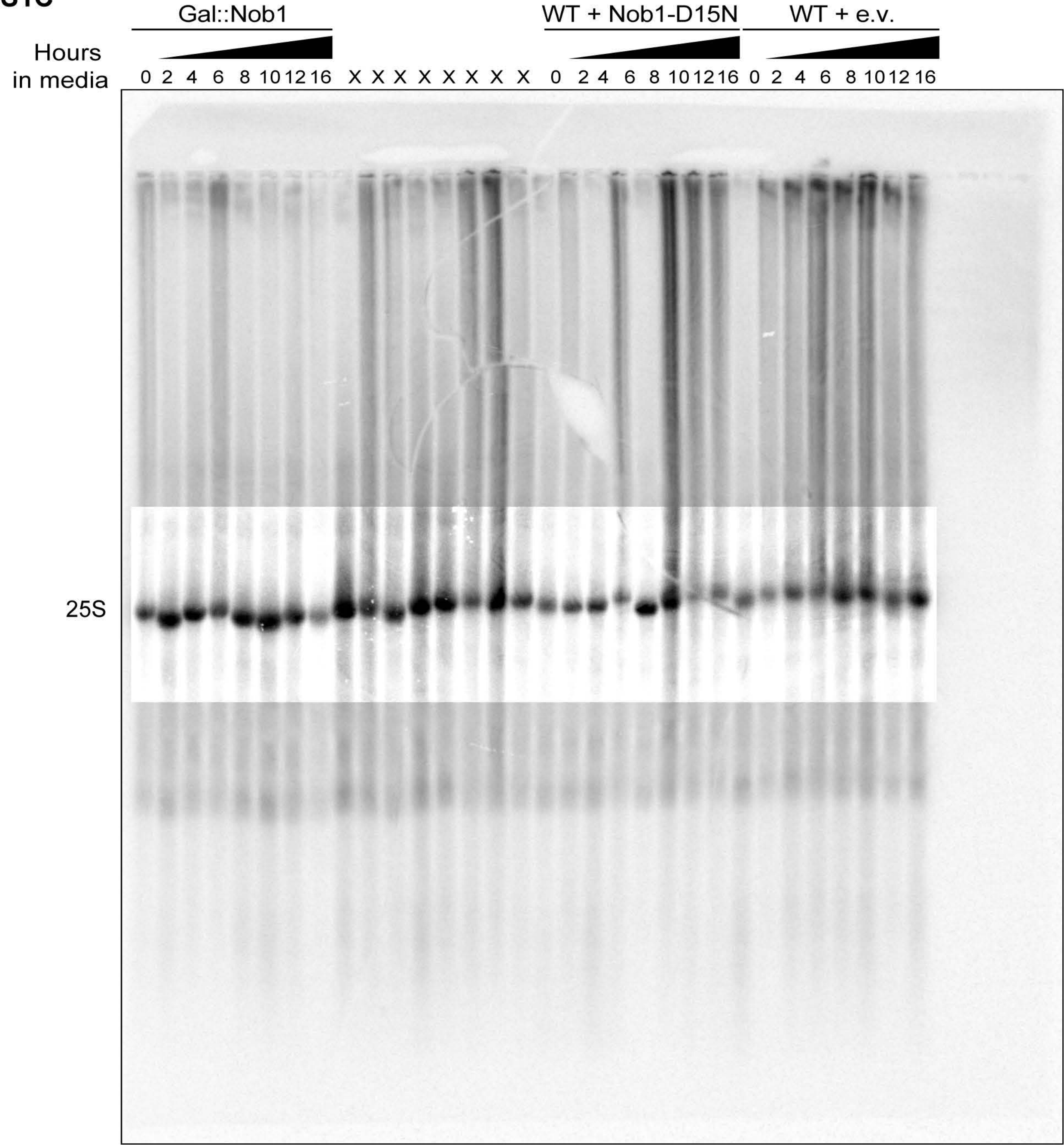
Gal::Nob1
WT + Nob1-D15N
WT + e.v.
Hours in media
0 2 4 6 8 10 12 16 X X X X X X X X 0 2 4 6 8 10 12 16 0 2 4 6 8 10 12 16
25S

S1C

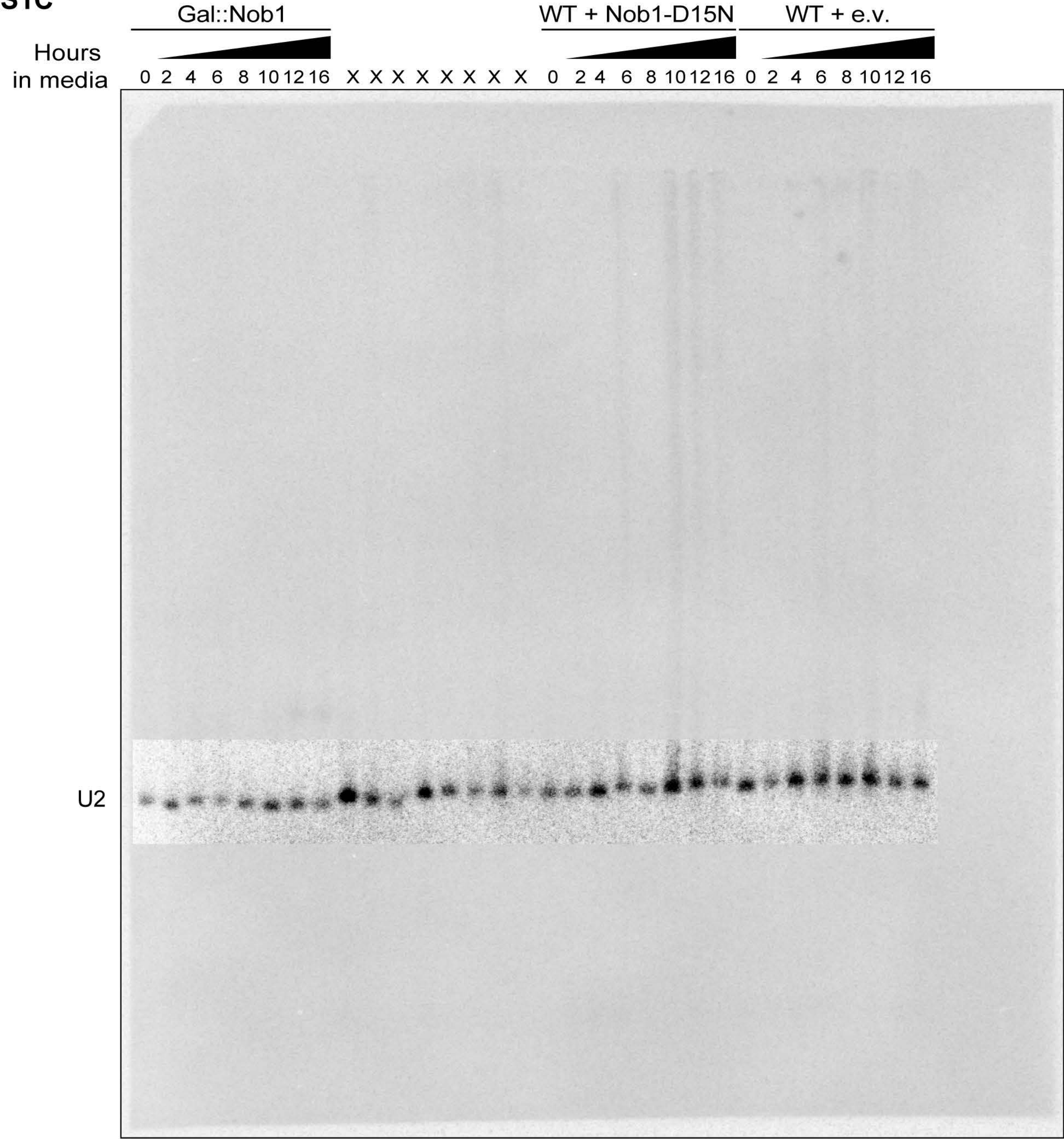

Gal::Nob1
WT + Nob1-D15N
WT + e.v.
Hours in media
0 2 4 6 8 10 12 16
X X X X X X X X
0 2 4 6 8 10 12 16
0 2 4 6 8 10 12 16
U2

S1D

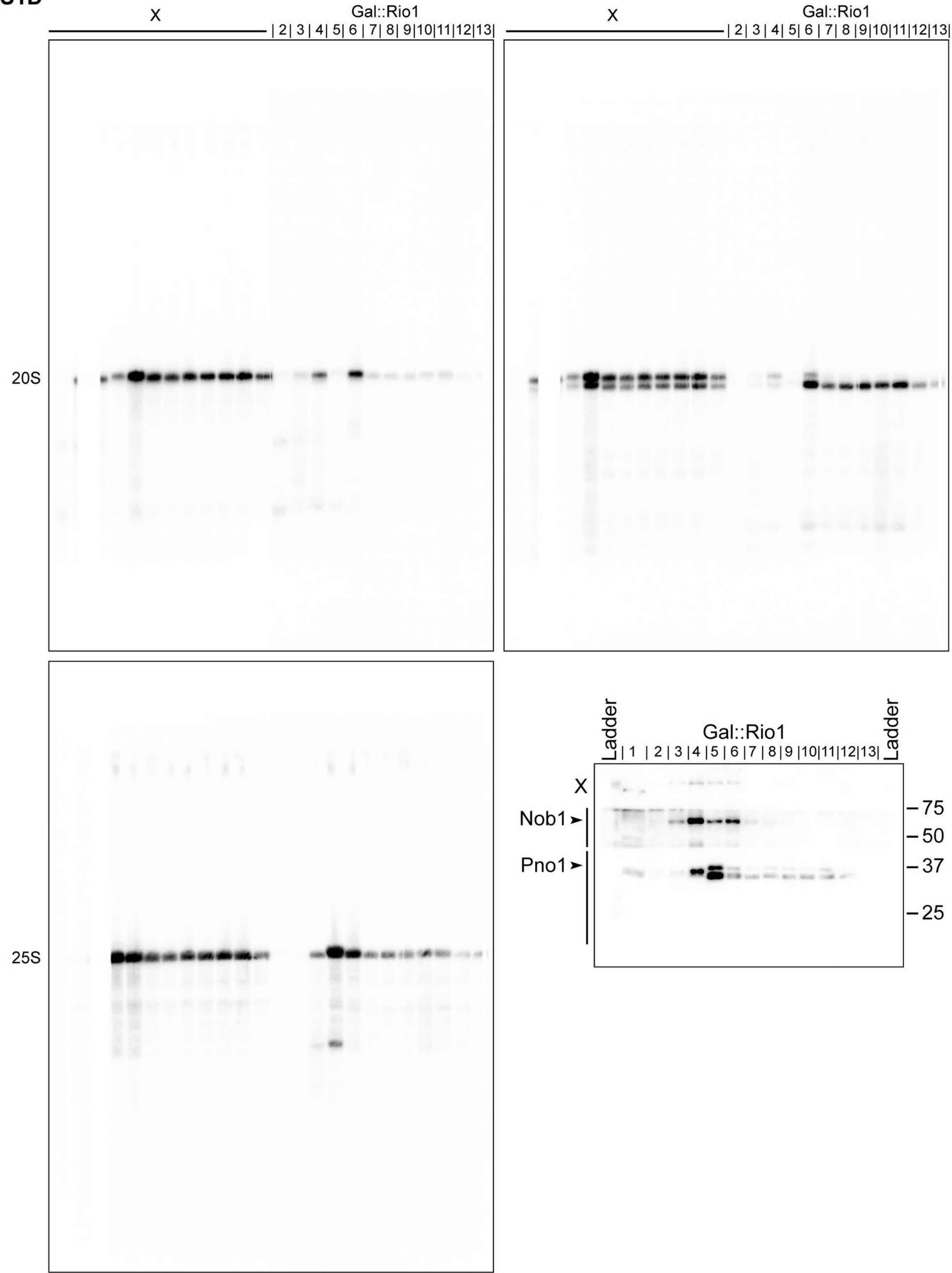

X
Gal::Rio1
| 2 | 3 | 4 | 5 | 6 | 7 | 8 | 9 |10|11|12|13|
20S
X
Gal::Rio1
| 2 | 3 | 4 | 5 | 6 | 7 | 8 | 9 |10|11|12|13|
20S
18S
25S
Ladder
Gal::Rio1
| 1 | 2 | 3 | 4 | 5 | 6 | 7 | 8 | 9 |10|11|12|13|
Ladder
X
Nob1
Pno1
75
50
37
25

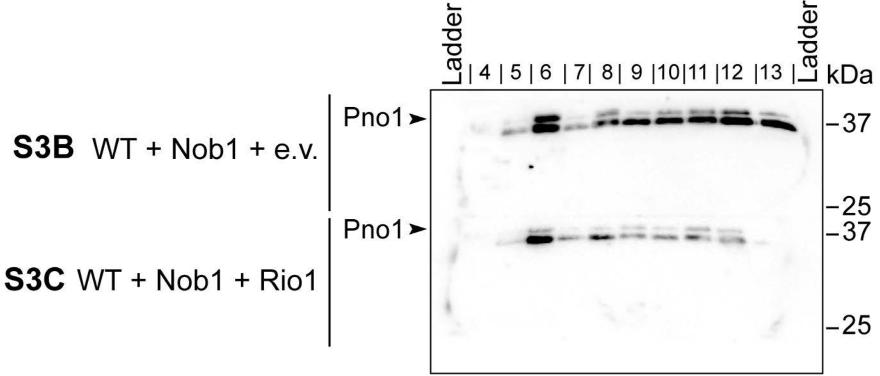
Ladder
| 4 | 5 | 6 | 7| 8| 9 |10|11 |12 |13 |
Ladder
kDa
S3B WT + Nob1 + e.v.
Pno1
37
25
Pno1
37
S3C WT + Nob1 + Rio1
25

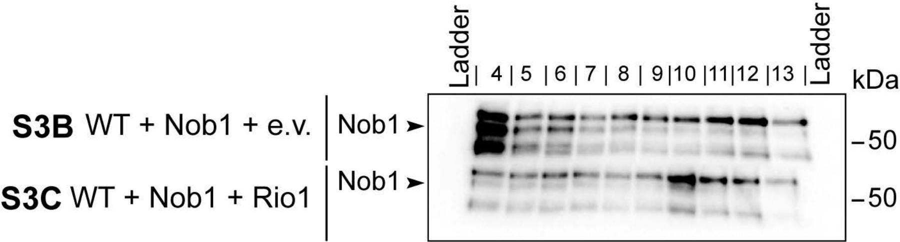
Ladder
| 4 | 5 | 6 | 7 | 8 | 9 |10 |11|12 |13 |
Ladder
kDa
S3B WT + Nob1 + e.v.
Nob1
50
Nob1
S3C WT + Nob1 + Rio1
50

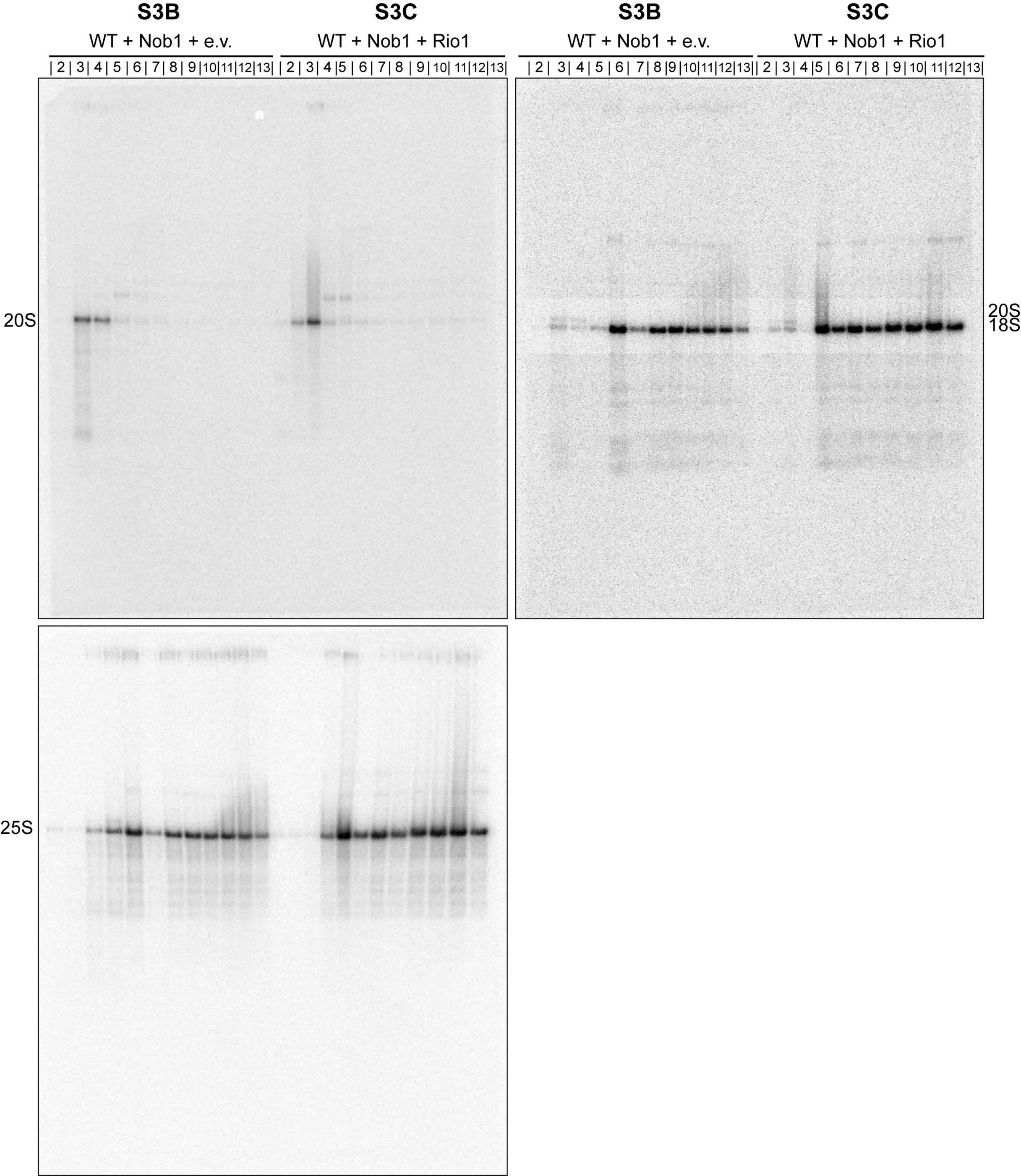
S3B
WT + Nob1 + e.v.
S3C
WT + Nob1 + Rio1
2 3 4 5 6 7 8 9 10 11 12 13
2 3 4 5 6 7 8 9 10 11 12 13
20S
18S
25S

S4A

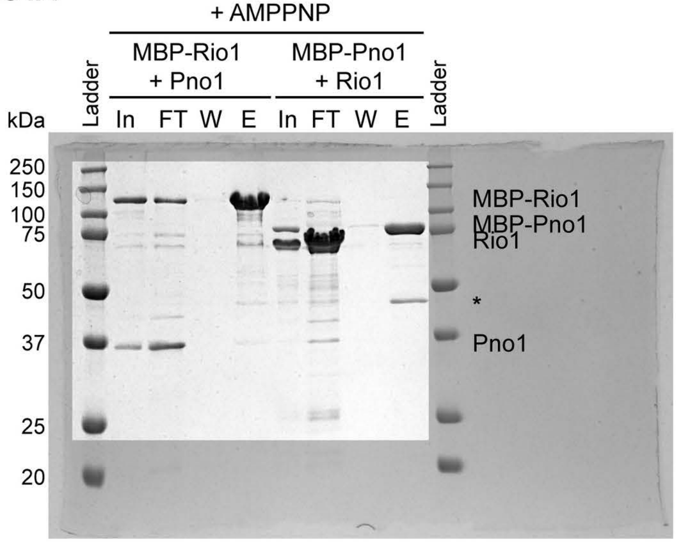

S4A

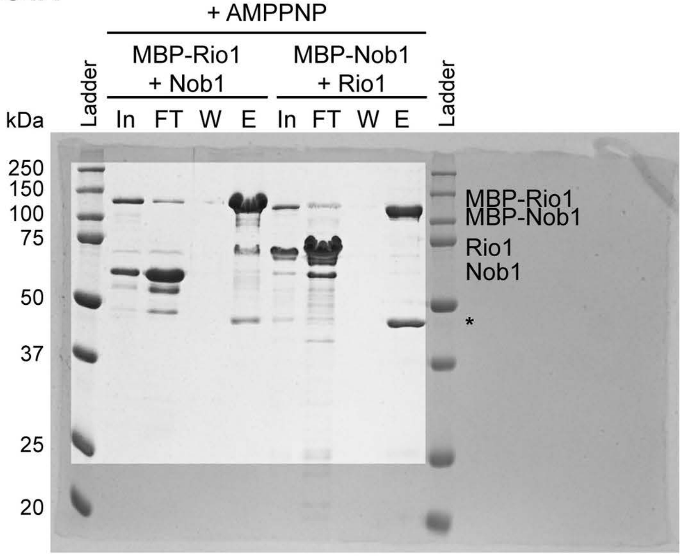

S4B

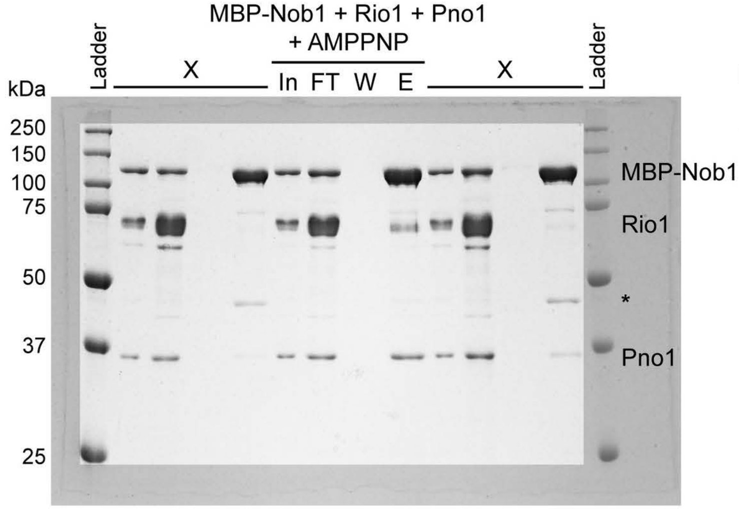

S4B

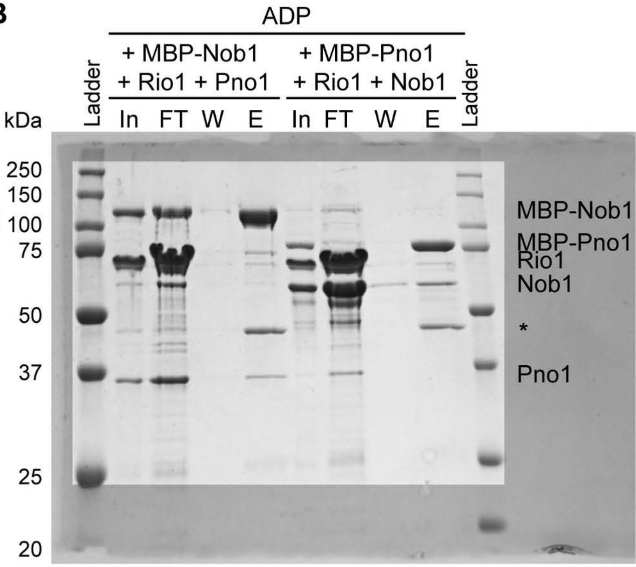

S4B

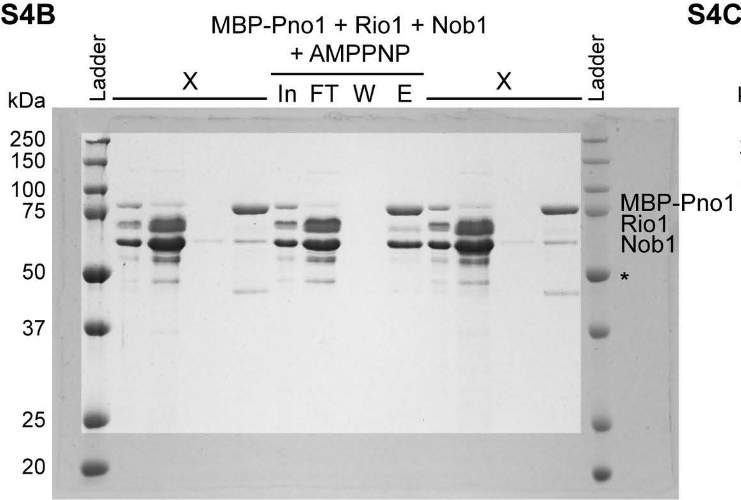

S4C

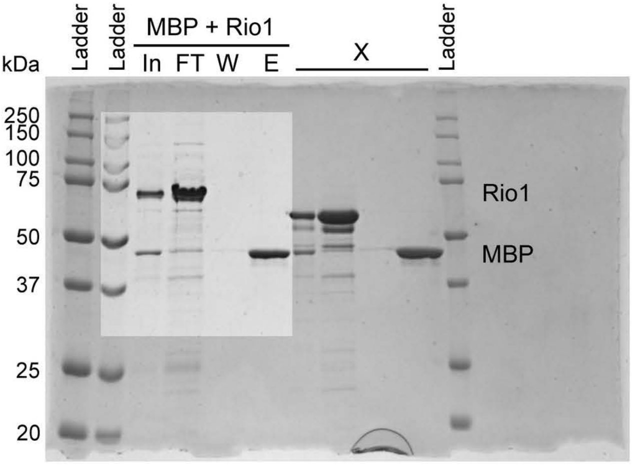

Supplement: S1 Raw Images — (PDF) [file pbio.3000329.s011.pdf]
