# Supplementary material for: A kinase-dependent checkpoint prevents escape of immature ribosomes into the translating pool
Source: PLoS Biol. 2019 Dec 13;17(12):e3000329. doi: 10.1371/journal.pbio.3000329 (PMC6934326; doi:10.1371/journal.pbio.3000329)
Supplement: Attachment — Submitted filename: Parker-Karbstein NS RG2.docx [file pbio.3000329.s012.docx]

Parker et al. use yeast genetics and biochemical reconstitution to provide evidence for how three late-stage 40S ribosome assembly factors (AFs) coordinate to establish a quality-control checkpoint that prevents release of immature 40S subunits into the active translational pool. While the study provides an exciting new model with some compelling data, the challenges for the study are the methods used for quantitation in many places (“quantitated” western blots which are notoriously problematic) and the relatively modest magnitude of effects. The strengths of the study are the use of yeast genetics as a method to identify roles for critical factors and the coupling with ribosome biochemistry. The section at the end focusing on translational defects associated with the RPS26-deficient ribosomes was particularly weak and added no mechanistic insight. Overall, the authors do provide new potential insights into a Rio1- and Nob1-mediated checkpoint that prevents release of immature ribosomes into the translating pool, though more rigorous quantitative approaches are needed to support this new model.

Systematic comments:

The authors initially show that in yeast depleted of the endonuclease Nob1, immature 40S subunits containing unprocessed 20S rRNA (and Nob1’s binding partner, Pno1) enter the active translational pool. However, a catalytically inactive dominant-negative Nob1 (D15N) prevents escape of such immature ribosomes, suggesting that Nob1 blocks mRNA recruitment and entry of immature ribosomes into the translational pool. This is a striking and interesting lead for the manuscript.

Comment: *This result is initially confusing and could be better discussed at this early stage – if Pno1 is present then these immature ribosomes presumably lack RPS26 and yet are still translating (this connects with earlier work from this laboratory). Given this striking phenotype, this would be an appropriate place to mention that while these immature ribosomes translate, they most likely suffer from translational defects (as eventually characterized by reporter assays, Fig 5E). Additionally, these are clearly very sick cells where few polysomes are formed. Do the authors check whether ribosome levels, global protein amounts and/or homeostasis is perturbed in ΔNob1 strains? Is the UPR (characterized by eIF2α phosphorylation) upregulated in ΔNob1 strains? If so, can the phenotype be rescued by Nob1-D15N expression in the ΔNob1 background?*

Next, the authors hypothesize that cleavage of rRNA by Nob1 is a prerequisite for its release from immature 20S-rRNA-containing ribosomes. To address this question, the authors use gel-shift assays to show that Nob1 binds its 20S substrate-mimic (H44-A2) and 3’ cleavage product (D-A2) more efficiently compared to the 18S mature rRNA product-mimic (H44-D).

Comment: *The binding affinities reported for all substrates are in the high nM (low µM) range which seems weak for typical RNA-protein interactions. More importantly, there is a modest two-fold difference in binding affinity between the substrate (H44-A2) and mature product (H44-D) mimics. Given this modest difference in affinity, the authors should tone down their statements when stating differences in these relative binding affinities (Page 13 “…binding affinities for the precursor rRNA and ITS1 product are indistinguishable, and much stronger than for the mature 18S rRNA product.”*

Next, the authors performed a clever overexpression (OE) screen to identify candidates that rescue the dominant-negative growth phenotype of Nob1-D15N and identified the kinase Rio1 as rescuing the D15N growth phenotype; the growth phenotype was very convincing for this experiment. The authors go on to try to show that Rio1 OE rescues the D15N phenotype by releasing immature 20S pre-rRNA containing ribosomes into the translating pool by quantitating 20S rRNA in the fractions across a sucrose gradient.

Comment: *The RNA analysis for Figure 3 was not compelling. While the authors are clearly able to run gels that nicely distinguish between 18S and 20S RNAs, these products were not sufficiently resolved in Figure 3B, nor was the decision to quantify relative to U2 totally obvious to me. The sucrose gradient is the better experiment, but here the effects are relatively modest (30% vs. 53%) though the exposures seem to have been deliberately chosen to make the result look stronger than the quantitation reveals. Fundamentally, the dominant negative is not that potent, and so the background is high for the experiment. At a minimum, these experiments should be performed such that the RNA products are well resolved.*

The authors performed pulldown assays to show that MBP-Rio1 binds the Nob1•Pno1 complex in the presence of non-hydrolysable ATP.

Comment: *While these experiments are well-controlled showing that Rio1 specifically binds the Nob1•Pno1 complex, but not Nob1 and Pno1 individually, based on the data it is impossible to interpret/conclude whether Rio1 recognizes the Nob1•Pno1 interface – the pulldowns in Figure 4A clearly show that Nob1 and Pno1 are not stoichiometric - the conclusion is overstated.* *If the authors wish to claim that Rio1 recognizes the Nob1•Pno1 interface, they should perform cross-linking MS analyses to determine residues at the binding interface based off distance constraints imposed by the cross-linker.*

The authors set up a pelleting assay using ribosomes purified from TAP-tagged Pno1-ΔRio1 strains to show that addition of recombinant Rio1 and ATP results in release of Nob1 and Pno1 from ribosomes.

Comment: *Although these are likely difficult experiments, the release phenotypes for Nob1 and Pno1 with Rio1 and ATP are very weak (this could stem from suboptimal reaction conditions, incomplete reaction time-course, or limited Rio1 enzymatic activity). Moreover, western blots are semi-quantitative and so this experiment does not strongly support the prediction that Rio1 dissociates Nob1 and Pno1. The results further indicate that ~20% Nob1 is released the presence of ADP (compared to 40% with ATP). Since the background is again high for this experiment, the authors should compare WT- Rio1 with the kinase and ATP-binding mutants to test if they can get a stronger phenotype.*

Next, the authors screened for weak-ribosome binding mutants for Nob1 and Pno1 that rescue the growth-defect imposed by ΔRio1, and identified Pno1-KKKF and Nob1-1-363 as mutants/deletions that rescue the ΔRio1 phenotype and enable immature ribosomes to enter the translational pool. The authors further hypothesize that the immature 20S rRNA and Pno1-containing ribosomes would lack RPS26 due to binding-interface incompatibility, and therefore tested whether RPS26 is sub-stoichiometric in ribosomes purified from Pno1-KKKF strains (compared to Pno1-WT strains). To perform this analysis, they chose 5 different r-proteins for normalization.

Comment: *While RPS26 appears to be sub-stoichiometric compared to RPS0 and RPS2 in Pno1-KKKF strains, the ratio is identical when comparing RPS5, RPS10 and RACK1. The authors should elaborate on why they chose the specific RPs for comparison? As before, westerns blots are semi-quantitative making it hard to interpret the result. A better experiment would involve isolating polysomes from KKKF and WT strains, normalizing for equal ribosomal amounts, and quantitative mass-spectrometry. From this data, they could interpret whether the ratio of RPS26 compared to an array of other RPs is lower in the KKKF compared to WT strains. Normalizing for total ribosomes is important, because from the sucrose gradient profiles it is evident that Pno1-KKKF has increased 60S and decreased polysome populations, suggesting a global defect in ribosomal numbers.*

In the final section, the authors attempt to connect the S26 deficiency to earlier work from their group, arguing that these immature S26-deficient ribosomes are stress-tolerant and translationally perturbed using growth assays and luciferase reporters.

Comment: *The results section for Fig 5E (defects in translation) is poorly written, shows modest effects (≤1.5-fold), and there is no mechanistic insight that is revealed.*

*For example, it is the presise that ΔRPS26 and ΔNob1 strains should both have ribosomes that lack S26. However, their effects on miscoding, stop codon selection, and +1 FS do not phenocopy each other.*

*Pno1-KKKF should also generate ribosomes that lack RPS26 – fundamentally therefore, all three strains should have the same effects on translation, and this is clearly not the case. In some cases (start codon selection, miscoding and stop codon readthrough) only KKKF and ΔRPS26 phenocopy each other; in other cases (+1 FS) ΔNob1 and ΔRPS26 phenocopy each other. These data add little to the story and instead raise a series of questions that are not addressed.*
