# Supplementary material for: A kinase-dependent checkpoint prevents escape of immature ribosomes into the translating pool
Source: PLoS Biol. 2019 Dec 13;17(12):e3000329. doi: 10.1371/journal.pbio.3000329 (PMC6934326; doi:10.1371/journal.pbio.3000329)
Supplement: Attachment — Submitted filename: reviewers response2.docx [file pbio.3000329.s013.docx]

Editorial Summary:

In light of the reviews (below), we will not be able to accept the current version of the manuscript, but we would welcome resubmission of a much-revised version that takes into account the reviewers' comments. Our academic editor advises that reviewer 1’s request for Rio1 ATPase measurements +/- Nob1-Pno1 is reasonable and would provide support for your model that “Rio1 releases Nob1 and Pno1 from nascent ribosomes in an ATPase- dependent manner”. Reviewer 3 also requests additional evidence to support this claim. Specifically, you are suggested to repeat the Nob1/Pno1 ribosome release assays comparing WT Rio1 with kinase and ATP-binding mutants. Our academic editor believes that this is a good and reasonable suggestion. Reviewer 2 raises concern about the evidence for suppression of Rio1 depletion by Pno1-KKKF and requests an alternative genetic approach that is feasible. Reviewer 3 raises related concerns about the characterization of the Pno1-KKKF strain by semi-quantitative western blotting for various RPs. The proposed quantitative mass spec analysis is an expensive experiment, but one you have used previously in related work, so this suggestion is not unreasonable. Our academic editor agrees with reviewer 3 that the western blots are hard to interpret and suggests that relating the genetic interaction (between Rio1 depletion and Pno1-KKKF) to a molecular phenotype is important for the story; this is the main evidence supporting your claim that “bypassing Rio1 via self-releasing mutations in Pno1 results in release of immature ribosomes” into the translating pool where they malfunction.

Our academic editor indicates that although reviewer 3 proposes many additional experiments, most are presented as optional if you modify the text to remove strong claims from weak evidence. We are inclined to let you decide which of these experiments you want to do, and let the reviewer decide whether the text has been modified adequately in the revision.

Our response:

We would like to thank the reviewers and the editor for their fair and thorough reviews. We have carefully addressed the comments of the reviewers as detailed below. As a result, the manuscript now includes 2 additional Supplemental Figures, as well as several panels in the main manuscript.

We must note here that while we did carry out the suggested mass spec experiment, which confirmed depletion of Rps26, the data also indicated that other late-assembling ribosomal proteins are depleted. Because the mechanistic basis for this is unclear, and because this complicates the interpretation of any stress and fidelity phenotypes, we have left out this part from the manuscript, in keeping with reviewer 3’s comments that these “add little”. Importantly, our conclusion that immature ribosomes are released into the translating pool upon Rio1 bypass does not depend on this Western analysis, and indeed derives from the finding of 20S-pre-rRNA in the polysomes in the Nob1-363 and Pno1-KKKF cells (**Figure 5**).

Reviewer #1: In this manuscript Parker et al utilize yeast genetics, cell based assays, and in-vitro RNA binding and protein binding assays to further our understanding of the role of three essential ribosome assembly factors Nob1, Pno1, and Rio1 in the assembly of the small ribosomal subunit. Overall the work is novel, comprehensive, well done and supports the model that the Rio1-Nob1-Pno1 network safeguards cells from releasing immature 40S subunits into the pool of translating ribosomes. This manuscript is worthy of publication in PLOS Biology however I have a few concerns that need to be addressed.

1.	I disagree with the authors interpretation of Fig. 2. The difference in kd for the three different RNA substrates is very small. While I agree that binding is weaker once A2 is gone, I don’t understand how the authors can claim this signifies that Nob1 dissociates with A2 following cleavage. To truly make this type of claim the authors should carry out competitive binding assay with H44 and A2. Moreover as the authors point out in the manuscript cleavage alone is not sufficient for Nob1 release.

We agree with the reviewer that the differences in Nob1 affinity between these RNAs is small, and we have therefore adjusted our description of this part. This revised section tones down the wording, in keeping with the small differences in binding affinity, and also, together with the following paragraph, aims to clarify our main point: Nob1-dependent cleavage promotes its release, but alone is not sufficient for cleavage. Indeed, Rio1 is required in addition. Nonetheless, the RNA binding differences helps set up the checkpoint to promote temporal regulation after rRNA cleavage.

The revised manuscript reads as follows:

*rRNA cleavage facilitates Nob1 release*

*If Nob1 release from nascent 40S requires rRNA cleavage by Nob1, then Nob1 blocking mRNA recruitment to premature ribosomes would enable a quality control mechanism to ensure only ribosomes containing matured rRNA enter the polysomes. To test whether Nob1-dependent rRNA cleavage facilitates its dissociation from ribosomes, we used a previously described quantitative in vitro RNA binding assay (16). This assay measures the binding of Nob1 to mimics of the 20S pre-rRNA substrate (H44-A2), the 18S rRNA ribosome product (H44-D), or the 3’-ITS1 product (D-A2) via native gel-shift. The data show that Nob1 binds the substrate mimic and the 3’-ITS1 product with similar affinities (K_d_= 0.93 and 0.96 µM, respectively). In contrast, Nob1 binds the 18S rRNA mimic somewhat more weakly (K_d_ =1.89 µM) (****Figure*** ***2****). These differences, albeit small, suggest that Nob1 predominantly interacts with ITS1, consistent with previous structure probing data (16, 27). Furthermore, the data suggest that after Nob1 cleaves 20S pre-RNAs, Nob1 will remain bound to its 3’-cleavage product and not to the matured 18S rRNA. Thus, Nob1 blocks premature ribosomes from binding mRNA until rRNA is cleaved, which facilitates Nob1 dissociation from nascent ribosomes, thereby setting up a mechanism to ensure only ribosomes with fully matured rRNA enter the translating pool.*

2.	The authors nicely show that Nob1-Pno1 interact with Rio1 in an ATP dependent manner and that Rio1 can release Nob1-Pno1 from nascent 40S subunits in vitro. Together this data suggests that Nob1-Pno1 stimulates Rio1 ATP hydrolysis. The authors should measure rates of Rio1 ATP hydrolysis in the presence and absence of Nob1-Pno1. It would be nice but it is not essential if the authors could show that a Walker B mutant of Rio1 blocks Nob1-Pno1 release in their in vitro release assay.

As requested by the reviewer, we have measured the rate of ATP hydrolysis by Rio1 in the presence and absence of Nob1/Pno1. Addition of these proteins does not impact the rate of ATP hydrolysis by Rio1 as now shown in **Supplemental Figure S4D.**

In addition, we have tested the role of ATP hydrolysis by Rio1 in the release assay, using two mutants D244A and D261A. These mutate the aspartate that ligands the catalytic Mg, or the phosphoaspartate site, respectively. As expected, these block Rio1-dependent Nob1 and Pno1 release. This data is now included in **Figure 4B**.

3.	There appears to be some inconsistency with the Nob1 Western Blots. In contrast the Pno1 Westerns are much more consistent. Some Nob1 blots show a high degree of non-specificity and it’s unclear how the authors know which band corresponds to Nob1. For example in the Westerns in Figure S1, B has a single band, C has 3 bands and D has 2 bands. The exposure time of the Nob1 westerns also varies quite a bit from figure to figure. The authors should either repeat these westerns, provide an explanation (protein degradation perhaps?), and/or include the uncropped western blots as a supplemental figure.  

The reviewer raises an issue that we have been trying to fight for a long time. Nob1 has two degradation products, which are the lower bands. We have mapped these and in fact Nob1_363 (Figures 5& S6) is the smallest one of these. The Nob1 Westerns look good, when we probe endogenous Nob1 (**Figure S1D&data not shown**), or Nob1_363 (**Figure 5A**). Under those conditions the full-length Nob1 is the predominant species. However, when we overexpress Nob1, especially under the strong Gal promoter (**Figure 1 D,E&F, 3C, S3B&C),** then the Westerns look not so nice, and the multiple bands appear. We think this is because on the pre-40S Nob1 is a lot more stable than off the pre-40S, and excess Nob1 by definition is mostly unbound. In contrast, most endogenous Nob1 is pre-40S-bound (Figure S1D), and therefore stable. Nob1_363 is the stable degradation product, and therefore no longer prone to degradation.

We are also including the uncropped Westerns in supplemental data.

Minor Concerns:

1.	In Fig. 4A the contrast level of the gel is too high, making it very hard to see the band for Pno1.

We have adjusted the contrast in Figure 4A.

2.	The model in Fig. 6 is a little hard to follow. I would suggest that the authors split the top and bottom into two separate panels, so they can distinguish WT-Pno1 from Pno1-KKKF.

We have modified the Figure as suggested by the reviewer.

Reviewer #2: The work by Parker and coworkers establishes an important quality control checkpoint of the final step of 40S subunit biogenesis. The work focusses on the role of the nuclease Nob1, required for the processing of 20S to 18S rRNA, the Nob1 binding partner Pno1, and the atypical kinase Rio1. Involvement of these factors in 40S maturation was previously reported. The authors here confirm some of the earlier findings, e.g. that Rps26 is assembled into 40S subunits late, after removal of Pno1. In addition the work provides novel insight into how the last steps of 40S maturation allow for proper quality control. The data reveal that 20S rRNA processing is required for the release of Pno1 and Nob1 from pre-40S particles. The data show that Rps26 only assembles after Pno1 release and rRNA processing, by that marking 40S subunits as "functional". In addition the release of Pno1/Nob1 requires the activity of Rio1 kinase. Interestingly, Rio1 requirement (Rio1 is normally essential) can be overcome my mutations within Pno1, which destabilize ribosome-binding of Pno1. The data reveal the such mutations within Pno1 result in the production of translating ribosomes, containing 20S rRNA and lacking Rps26. The data further suggest that Rio1-independent release of Pno1 mutants, with ribosome-binding defects rescues the lethality of Rio1.

The presented data are mostly convincing, clearly presented, and justify the conclusions drawn. I have a few comments for the authors.

My only major concern is that suppression of Rio1-depletion by Pno1-KKKF is not that convincing. In Fig. 4C is looks fine, however, in Fig. S3a I can hardly see a difference e.g. between the effect of Pno1-KKKF and Pno1-DDD/K (and also Pno1 wild type ...).The GAL depletion system has its problems. I suggest to show rescue in a more stringent way: use a diploid heterozygous Drio1Dpno1 strain, transform with the wild type Pno1 or the relevant mutants on a plasmid, dissect, and show that the strain Drio1Dpno1 + plasmid-borne Pno1-KKKF is viable. This is doable and would significantly strengthen one of the major conclusions of this work.

We have redone the panel in Figure S3A (now Figure S5A), where the previous image was just taken too early. The repeated assay, together with the panel in the main text, shows that indeed the KKKF mutant provides for significant rescue (especially given its substantial phenotype in the presence of Rio1).

In addition to repeating the serial dilutions, we have also carried out quantitative growth measurements. These are now shown as **Figure S5B,** and also demonstrate that (i) KKKF partially rescues the absence of Rio1 and (ii) that KKKF is largely immune to depletion of Rio1.

We were unable to carry out the sporulation assay, as we have no expertise in doing that. There is no other yeast lab at Scripps Florida or the neighboring Florida Atlantic University, and we do not even have a dissection scope to separate spores. Nonetheless, we feel that the two additional assays show conclusively that the lethal phenotype from Rio1 depletion is rescued in the presence of the Pno1-KKKF mutant.

In this context. Rio1 was found to act also in other cellular processes. This should be discussed also with respect to the complementation of Drio1 by Pno1-KKKF. Do the authors think that the 40S maturation function is the only essential function of Rio1?

The reviewer brings up an interesting point that we avoided due to the controversy it is sure to stir. Nonetheless, s/he is certainly correct in interpreting the KKKF rescue to suggest that the only essential function of Rio1 is in 40S maturation. In this regard it is similar to Hrr25. As requested, this is now discussed in the manuscript and reads as follows:

*“Previous work has also established roles for Rio1 in cell division, where is binds the rDNA locus, interacts with the RENT complex and the helicase Sgs1, which both regulate rDNA silencing, the stability of the locus, and its condensation during cell segregation. Accordingly, Rio1-depleted cells have defects in these processes (50, 51). The rescue of cell viability in the absence of Rio1 by the Pno1-KKKF mutant indicates that the essential role of Rio1 is in ribosome assembly. However, the small but significant difference in doubling times of cells containing Pno1-KKKF with and without Rio1 (****Figure S5B)*** *would be consistent with additional (non-essential) roles for Rio1 outside of ribosome assembly.”*

Page 6: The observed differences with respect to the Kds are rather moderate. I would not over interpret these data.

As described in the response to reviewer 1, we have toned down the interpretation of these data. The revised section now reads as follows:

*rRNA cleavage facilitates Nob1 release*

If Nob1 release from nascent 40S requires rRNA cleavage by Nob1, then Nob1 blocking mRNA recruitment to premature ribosomes would enable a quality control mechanism to ensure only ribosomes containing matured rRNA enter the polysomes. To test whether Nob1-dependent rRNA cleavage facilitates its dissociation from ribosomes, we used a previously described quantitative *in vitro* RNA binding assay (16). This assay measures the binding of Nob1 to mimics of the 20S pre-rRNA substrate (H44-A2), the 18S rRNA ribosome product (H44-D), or the 3’-ITS1 product (D-A2) via native gel-shift. The data show that Nob1 binds the substrate mimic and the 3’-ITS1 product with similar affinities (*K*_d_= 0.93 and 0.96 µM, respectively). In contrast, Nob1 binds the 18S rRNA mimic somewhat more weakly (*K*_d_ =1.89 µM) (**Figure** **2**). These differences, albeit small, suggest that Nob1 predominantly interacts with ITS1, consistent with previous structure probing data (16, 27). Furthermore, the data suggest that after Nob1 cleaves 20S pre-RNAs, Nob1 will remain bound to its 3’-cleavage product and not to the matured 18S rRNA. Thus, Nob1 blocks premature ribosomes from binding mRNA until rRNA is cleaved, which facilitates Nob1 dissociation from nascent ribosomes, thereby setting up a mechanism to ensure only ribosomes with fully matured rRNA enter the translating pool.

Page 7: Please indicate what is meant by a "limited screen" and which factors were tested in this screen.

In this screen we have also tested overexpression of Fap7, Hrr25, and Rio2. These data are now shown as Supplemental Figure **S2**.

Page 8: "Rio1 phosphorylation functions as a switch, which is reset after hydrolysis". I did not understand this sentence. Please include 2-3 sentences, explaining the current model (or the model of the authors!) of how Rio1 is supposed to affect release of Pno1/Nob1. Rio1 seems to bind in the ATP-bound state. And then? In this context: "kinase-dependent" (in the Title of the work) to the general reader suggests that some phosphorylation event is involved in the process. Is this the idea? Or do the authors think that it is rather ATP hydrolysis by which Rio1 drives the process? Please provide some mechanistic ideas (even if speculative).

We have rephrased the sentence the reviewer is referring to as follows:

*“By analogy to its close relative, Rio2, it is believed that the Rio1 functional cycle involves ATP binding, autophosphorylation, and subsequent dephosphorylation, resulting in net ATP hydrolysis, which must be coupled to conformational changes in Rio1 or its binding partner(s) (Ferreira-Cerca et al., 2012; Knuppel et al., 2017).”*

In addition, the discussion contains this speculation about the Rio1 mechanism:

*“Rio1 is an atypical aspartate kinase. Analogous to its close relative, Rio1 is believed to use a cycle of autophosphorylation and subsequent dephosphorylation to promote its function in 40S ribosome biogenesis (45, 46). Our binding data indicate that ATP-bound Rio1 binds ribosome-bound Nob1•Pno1. Furthermore, the release data show that dissociation of the complex requires phosphoryl transfer. We thus speculate that phosphorylation of Rio1 (and presumably release of the ADP) are required to promote a conformational change, which leads to release of Nob1 and Pno1, with the cycle being re-set by Rio1 dephosphorylation.”*

The work by Schutz et al 2014 suggested that Rps26 is required for cytoplasmic processing of 20S pre-rRNA to mature 18S rRNA. Please discuss these findings in the light of your data.

The referenced manuscript by Schutz suggests that Rps26 is incorporated into the earliest 40S precursor, the 90S processosome. There is wide agreement between many labs that that is not the case, based on (i) the absence of Rps26 in 90S structures from three different labs, as well as (ii) the much later cytoplasmic pre-40S structures from 5 different labs. This is explained by the binding of Krr1 (early) and Pno1 (later) to the site later occupied by Rps26 (Strunk et al, Science 2011; Heuer et al., Elife, 2017; Sturm, Nature Communications 2017). In addition, (iii) Ferreira-Cerca et al. (Mol Cell 2007) have shown a number of years ago that Rps26 is the only tested (22/32) 40S ribosomal protein that does not precipitate the 20S rRNA precursor, as expected if it doesn't bind pre-rRNA until after maturation. (iv) Finally, we have shown that 40S ribosomes lacking Rps26 are fully mature (Ferretti et al., *NSMB* 2017).

The observation that 20S rRNA accumulates upon depletion of Rps26 is explained by a surprise observation we made when studying the mRNA preference of Rps26-deficient (but otherwise mature) 40S subunits: While the vast majority of AFs is either unaffected by Rps26 depletion or even preferred, the essential AF Fap7 is preferentially translated by mature Rps26-containing ribosomes (Figure S3i of Ferretti et al., 2017). Thus, when Rps26-deficient ribosomes accumulate, Fap7 is depleted, leading eventually to the accumulation of an earlier assembly intermediate, which contains 20S rRNA. We have included such a discussion in the manuscript as follows:

“Rps26 incorporation requires Pno1 release because Pno1 blocks its binding to pre-40S ribosomes (19, 20). Previous data have shown that Rps26 depletion leads to accumulation of 20S rRNA (52), suggesting that Rps26 would be incorporated prior to rRNA maturation, not after, as suggested by the data herein. These data can be reconciled by the surprise observation that the fully matured 18S rRNA-containing but Rps26-depleted ribosomes that accumulate upon Rps26 depletion do not efficiently translate the late 40S assembly factor Fap7 (42). Thus, Rps26 depletion affects 20S maturation indirectly, by blocking the production of Fap7, leading to the accumulation of 20S rRNA-containing 40S assembly intermediates. This model, which reconciles the data herein with the data from Schutz et al., is further supported by the observation that high copy Fap7 is a suppressor of reduced amounts of Rps26 (53).”

The probes used to detect 18S and 20S rRNA species should be given in the Supplemental material.

The sequence of Northern probes is now indicated in the Methods section.

Reviewer #3: Parker et al. use yeast genetics and biochemical reconstitution to provide evidence for how three late-stage 40S ribosome assembly factors (AFs) coordinate to establish a quality-control checkpoint that prevents release of immature 40S subunits into the active translational pool. While the study provides an exciting new model with some compelling data, the challenges for the study are the methods used for quantitation in many places (“quantitated” western blots which are notoriously problematic) and the relatively modest magnitude of effects. The strengths of the study are the use of yeast genetics as a method to identify roles for critical factors and the coupling with ribosome biochemistry. The section at the end focusing on translational defects associated with the RPS26-deficient ribosomes was particularly weak and added no mechanistic insight. Overall, the authors do provide new potential insights into a Rio1- and Nob1-mediated checkpoint that prevents release of immature ribosomes into the translating pool, though more rigorous quantitative approaches are needed to support this new model.

Systematic comments:

The authors initially show that in yeast depleted of the endonuclease Nob1, immature 40S subunits containing unprocessed 20S rRNA (and Nob1’s binding partner, Pno1) enter the active translational pool. However, a catalytically inactive dominant-negative Nob1 (D15N) prevents escape of such immature ribosomes, suggesting that Nob1 blocks mRNA recruitment and entry of immature ribosomes into the translational pool. This is a striking and interesting lead for the manuscript.

Comment: This result is initially confusing and could be better discussed at this early stage – if Pno1 is present then these immature ribosomes presumably lack RPS26 and yet are still translating (this connects with earlier work from this laboratory). Given this striking phenotype, this would be an appropriate place to mention that while these immature ribosomes translate, they most likely suffer from translational defects (as eventually characterized by reporter assays, Fig 5E). Additionally, these are clearly very sick cells where few polysomes are formed.

We have revised this section in keeping with comments from this reviewer.

Do the authors check whether ribosome levels, global protein amounts and/or homeostasis is perturbed in ΔNob1 strains? Is the UPR (characterized by eIF2α phosphorylation) upregulated in ΔNob1 strains? If so, can the phenotype be rescued by Nob1-D15N expression in the ΔNob1 background?

Nob1 is essential for 40S ribosome assembly. Thus, over an extended time, the amount of 40S ribosomes in those strains will be reduced, which is reflected in the large peak of free 60S in Figure 1A. Nonetheless, the polysomes in that strain are the most normal to any of the dozens of 40S assembly factors we have investigated, and nearly indistinguishable from wild type cells (*e.g.*, Figure 1A&D and Figure S3 B&C). Note that these cells were depleted for Nob1 for 12h.

The data in the manuscript show defects in start-site recognition from ΔNob1. As suggested, we have now extended this work and tested the resistance to specific stresses and found that ΔNob1 cells are resistant to caffeine and high salt, different from ΔRps26 cells, which are not resistant to caffeine. These data are now shown as Figure 1C. They suggest a perturbation in translation from Nob1 deficiency.

As also requested, we have also probed whether eIF2α is phosphorylated in the ΔNob1 background. This does not seem to be the case. These data are shown as Figure S1A.

We have rewritten the introduction taking into account the suggestions from the reviewer and hope that s/he finds this revised version easier to follow, and sufficiently emphasizing what s/he calls our “striking lead”. We would certainly welcome any additional specific comments on the presentation.

Next, the authors hypothesize that cleavage of rRNA by Nob1 is a prerequisite for its release from immature 20S-rRNA-containing ribosomes. To address this question, the authors use gel-shift assays to show that Nob1 binds its 20S substrate-mimic (H44-A2) and 3’ cleavage product (D-A2) more efficiently compared to the 18S mature rRNA product-mimic (H44-D). 

Comment: The binding affinities reported for all substrates are in the high nM (low µM) range which seems weak for typical RNA-protein interactions. More importantly, there is a modest two-fold difference in binding affinity between the substrate (H44-A2) and mature product (H44-D) mimics. Given this modest difference in affinity, the authors should tone down their statements when stating differences in these relative binding affinities (Page 13 “…binding affinities for the precursor rRNA and ITS1 product are indistinguishable, and much stronger than for the mature 18S rRNA product.”

As described in the response to reviewer 1, we have toned down the interpretation of these data. The revised section now reads as follows:

*“The data show that Nob1 binds the substrate mimic and the 3’-ITS1 product with similar affinities (K_d_= 0.93 and 0.96 µM, respectively). In contrast, Nob1 binds the 18S rRNA mimic somewhat more weakly (K_d_ =1.89 µM) (****Figure*** ***2****). These differences, albeit small, suggest that Nob1 predominantly interacts with ITS1, consistent with previous structure probing data (Lamanna and Karbstein, 2009; Turowski et al., 2014).”*

Next, the authors performed a clever overexpression (OE) screen to identify candidates that rescue the dominant-negative growth phenotype of Nob1-D15N and identified the kinase Rio1 as rescuing the D15N growth phenotype; the growth phenotype was very convincing for this experiment. The authors go on to try to show that Rio1 OE rescues the D15N phenotype by releasing immature 20S pre-rRNA containing ribosomes into the translating pool by quantitating 20S rRNA in the fractions across a sucrose gradient.

Comment: The RNA analysis for Figure 3 was not compelling. While the authors are clearly able to run gels that nicely distinguish between 18S and 20S RNAs, these products were not sufficiently resolved in Figure 3B, nor was the decision to quantify relative to U2 totally obvious to me. The sucrose gradient is the better experiment, but here the effects are relatively modest (30% vs. 53%) though the exposures seem to have been deliberately chosen to make the result look stronger than the quantitation reveals. Fundamentally, the dominant negative is not that potent, and so the background is high for the experiment. At a minimum, these experiments should be performed such that the RNA products are well resolved.

We have repeated this experiment and included a new Northern for total cells in Figure 3. This has not changed the conclusions.

The authors performed pulldown assays to show that MBP-Rio1 binds the Nob1•Pno1 complex in the presence of non-hydrolysable ATP.

Comment: While these experiments are well-controlled showing that Rio1 specifically binds the Nob1•Pno1 complex, but not Nob1 and Pno1 individually, based on the data it is impossible to interpret/conclude whether Rio1 recognizes the Nob1•Pno1 interface – the pulldowns in Figure 4A clearly show that Nob1 and Pno1 are not stoichiometric - the conclusion is overstated. If the authors wish to claim that Rio1 recognizes the Nob1•Pno1 interface, they should perform cross-linking MS analyses to determine residues at the binding interface based off distance constraints imposed by the cross-linker.

As requested, we have toned down the description of these data, and removed the conclusion about the interface. We also note, that while the complex is slightly substoichiometric, the intensity of coomassie staining is proportional to the size of the protein. Thus, Rio1-MBP is about 2 fold larger than Nob1, which is nearly 2 fold larger than Pno1. This accounts for most of the differences in the band intensity. The revised section is below.

*“To test this model, we performed in vitro protein binding assays with recombinant Rio1, Nob1, and Pno1. These experiments show that MBP-Rio1 binds Nob1 and Pno1, but not either Nob1 or Pno1 individually, suggesting that Rio1 recognizes the Nob1-Pno1 complex (****Figure 4A*** *and* ***S5****). Importantly, the presence of AMPPNP, a non-hydrolyzable ATP analog, is required for formation of the Rio1•Nob1•Pno1 complex, as little to no complex formation is observed in the presence of ADP (****Figure 4A*** *and* ***S4****).”*

The authors set up a pelleting assay using ribosomes purified from TAP-tagged Pno1-ΔRio1 strains to show that addition of recombinant Rio1 and ATP results in release of Nob1 and Pno1 from ribosomes.

Comment: Although these are likely difficult experiments, the release phenotypes for Nob1 and Pno1 with Rio1 and ATP are very weak (this could stem from suboptimal reaction conditions, incomplete reaction time-course, or limited Rio1 enzymatic activity). Moreover, western blots are semi-quantitative and so this experiment does not strongly support the prediction that Rio1 dissociates Nob1 and Pno1. The results further indicate that ~20% Nob1 is released the presence of ADP (compared to 40% with ATP). Since the background is again high for this experiment, the authors should compare WT- Rio1 with the kinase and ATP-binding mutants to test if they can get a stronger phenotype.

As requested, we have repeated this experiment with two Rio1 mutants, D244A and D261A. These mutate the aspartate that ligands the catalytic Mg, or the phosphoaspartate site, respectively. As expected, these block Rio1-dependent Nob1 and Pno1 release. These data are now included in the revised Figure 4.

Next, the authors screened for weak-ribosome binding mutants for Nob1 and Pno1 that rescue the growth-defect imposed by ΔRio1, and identified Pno1-KKKF and Nob1-1-363 as mutants/deletions that rescue the ΔRio1 phenotype and enable immature ribosomes to enter the translational pool. The authors further hypothesize that the immature 20S rRNA and Pno1-containing ribosomes would lack RPS26 due to binding-interface incompatibility, and therefore tested whether RPS26 is sub-stoichiometric in ribosomes purified from Pno1-KKKF strains (compared to Pno1-WT strains). To perform this analysis, they chose 5 different r-proteins for normalization.

Comment: While RPS26 appears to be sub-stoichiometric compared to RPS0 and RPS2 in Pno1-KKKF strains, the ratio is identical when comparing RPS5, RPS10 and RACK1. The authors should elaborate on why they chose the specific RPs for comparison?  As before, westerns blots are semi-quantitative making it hard to interpret the result. A better experiment would involve isolating polysomes from KKKF and WT strains, normalizing for equal ribosomal amounts, and quantitative mass-spectrometry. From this data, they could interpret whether the ratio of RPS26 compared to an array of other RPs is lower in the KKKF compared to WT strains. Normalizing for total ribosomes is important, because from the sucrose gradient profiles it is evident that Pno1-KKKF has increased 60S and decreased polysome populations, suggesting a global defect in ribosomal numbers.

We originally normalized against ribosomal proteins for which antibodies are available. We did indeed carry out the suggested experiment, and the results suggest that while ribosomes from Pno1-KKKF cells are deficient in Rps26, they are also deficient in a few other late-assembling ribosomal proteins from the head. Because deficiency of each of these proteins (or all of them together) could be responsible for the observed phenotypes on translational fidelity and stress resistance, we decided to remove these data. Furthermore, how premature release of Pno1 blocks assembly of these proteins is also completely unclear and not part of the story, which is about Rio1.

In the final section, the authors attempt to connect the S26 deficiency to earlier work from their group, arguing that these immature S26-deficient ribosomes are stress-tolerant and translationally perturbed using growth assays and luciferase reporters.

Comment: The results section for Fig 5E (defects in translation) is poorly written, shows modest effects (≤1.5-fold), and there is no mechanistic insight that is revealed.

For example, it is the presise that ΔRPS26 and ΔNob1 strains should both have ribosomes that lack S26. However, their effects on miscoding, stop codon selection, and +1 FS do not phenocopy each other. Pno1-KKKF should also generate ribosomes that lack RPS26 – fundamentally therefore, all three strains should have the same effects on translation, and this is clearly not the case. In some cases (start codon selection, miscoding and stop codon readthrough) only KKKF and ΔRPS26 phenocopy each other; in other cases (+1 FS) ΔNob1 and ΔRPS26 phenocopy each other. These data add little to the story and instead raise a series of questions that are not addressed.

As indicated above, we have removed this section, which the reviewer suggested “added little”. We did retain the fidelity data for Nob1 depleted cells and are presenting them at the beginning of the revised manuscript as suggested. We understand these better, as our data demonstrate that these ribosomes contain immature rRNA (in contrast to dRps26 ribosomes, which contain mature RNA), as well as Pno1. Even though Pno1 blocks Rps26 recruitment, its positioning could allow it to perform Rps26’s function on the ribosome, or an altered function (e.g. recognizing a G instead of an A at position -4). Thus, we do not expect the Nob1-depleted data to mirror Rps26-depletion data, and indeed they do not. They report on Pno1-containing ribosomes with 20S pre-rRNA, while Rps26-deficient ribosomes do not contain Pno1 or 20S rRNA, but mature 18S rRNA.
