# Supplementary material for: A kinase-dependent checkpoint prevents escape of immature ribosomes into the translating pool
Source: PLoS Biol. 2019 Dec 13;17(12):e3000329. doi: 10.1371/journal.pbio.3000329 (PMC6934326; doi:10.1371/journal.pbio.3000329)
Supplement: Attachment — Submitted filename: reviewerresponse2.docx [file pbio.3000329.s014.docx]

We would like to thank the reviewers for their careful and fair evaluation. We will address the remaining concerns voiced by reviewer 3 below.

Reviewer remarks:

Reviewer #1: Thank you for the opportunity to review this manuscript which establishes how three ribosome assembly factors safeguard cells from immature ribosomes. The authors have addressed all of my previous concerns. This manuscript is acceptable for publication in PLOS Biology.

Reviewer #3: The overall goal of this paper was to establish how three late-stage 40S biogenesis factors (Rio1, Nob1, and Pno1) coordinate to establish a QC checkpoint that prevents the release immature ribosomes into the translating pool. Overall, the authors have revised the manuscript, included new experiments, and addressed most queries that this reviewer had. This manuscript should certainly be published but there remain issues of clarity that could substantially increase the accessibility and dissemination of what has been discovered:

1.	It seems more reasonable to perform the Nob1-D15N OE experiment (Fig 1E) in the Gal::Nob1 (Fig. 1A) background. The absence of 20S rRNA in polysomes could be because of endogenous catalytically active Nob1 in these strains.

We apologize for not better understanding that this was the concern of the reviewer. However, we do not share this concern for the following reasons: Overexpressing Nob1-D15N in the background of wild type Nob1 produces a strong dominant growth defect (**Figure 3A and Figure S1B**) and leads to greater than 7-fold accumulation of 20S pre-rRNA (**Figure 3B**), demonstrating that Nob1 activity is strongly impaired (outcompeted) in these strains. Furthermore, comparing overexpression of wt Nob1 with overexpression of Nob1-D15N (**Figure 1D&E),** one can see that Nob1-D15N produces a phenotype: the accumulation of 80S-like ribosomes. In contrast, in cells with wild type Nob1, 40S precursors accumulate in the 40S fraction. These data further demonstrate that akin to Gal:Nob1, Nob1-D15N overexpression in the wild type background produces a defect in 18S rRNA maturation, but that the fate of the 40S precursors differs depending on the presence and absence of Nob1. Finally, the release of 20S pre-rRNA into the polysomes in the Nob1-D15N background is rescued by overexpressing Rio1, which does not affect Nob1 activity.

Together, these data strongly suggest that (i) the catalytic activity of Nob1 is impaired in the Nob1-D15N strain, even in the background of wild type Nob1, and that (ii) the observation that 20S pre-rRNA is not in the polysomes is not due to continued rRNA cleavage activity, but instead due to the presence of Nob1.

2.	Figure 3: The authors have not answered the reviewer’s concern. The reviewer is not concerned about the effect of 20S rRNA in total cells. What is important is that the release of 20S rRNA into polysomes by Rio1-OE in the Nob1-D15N-OE background is modest, and the gels seem to have been deliberately shown with different exposures (empty vector (3C,left), lower exposure; (3C, right) Rio1-OE, longer exposure) to make the phenotype look stronger than the quantitation reveal. To be honest, this remains a general problem in figures showing polysome profiles. As readers we are expected to look at difficult to quantitate westerns and northerns and to evaluate the author’s interpretation in the absence of any quantitation. I understand this is challenging to clarify but it really would be helpful. I really still am struggling in particular with figures 4 and 5 where effects are hard to see and there is no quantitation. Pno1 can be somewhat retained on deep polysomes but this is generally hard to see and not quantitated. Additionally, the model includes no indication that Pno1 is retained on ribosomes that are translating … but I think this is what the data suggest. Perhaps a short discussion in the “results” section of what the results might mean would be helpful. The data are shown, the strict interpretation stated, but I am struggling to fit what I am seeing into a developing model.

We are confused about these comments by the reviewer. It seems that he/she might be looking at the previous version of these Figures. Indeed, Figure 3C is an entirely unaltered image of one gel, which contains both samples run in parallel, transferred in parallel and exposed in parallel for the exact same time. Thus, these are directly comparable. The uncropped images in the supplemental data demonstrate this.

With regards to the comment about lack of quantification, all panels in Figures 4 and 5 that can be quantified were, with the exception of Figure 5A. We have added quantifications to that panel in the revised version of the manuscript.

Finally, the presence of Pno1 in the polysomes of Nob1-deficient cells is discussed in the discussion under the subheading “*Why do 20S pre-rRNA-containing ribosomes not support cell growth?”*

3.	For me, the discussion gets very much into the weeds and I am struggling to follow some of the main points, again, mostly connected to the final figures (4/5). It seems that the discussion presents an almost un-weighted summary of all literature, much of it contradictory, and so the strong points of the study get lost. The study makes strong claims and they should be easily deciphered in the final figure and in the summary in the discussion.

We have re-worked the discussion to streamline it. We do hope that it is easier to follow now.

4. Figures would be easier to interpret if the relevant information were found attached to each panel (so I don't need to dig into the legend) - for example Fig 4/5 polysomes are all Gal:Rio depleted, but this is not indicated on the actual panel.

We have added that information to the Figures.
